# Electrochemical patterns during *Drosophila* oogenesis: ion-transport mechanisms generate stage-specific gradients of pH and membrane potential in the follicle-cell epithelium

**DOI:** 10.1186/s12861-019-0192-x

**Published:** 2019-06-21

**Authors:** Isabel Weiß, Johannes Bohrmann

**Affiliations:** RWTH Aachen University, Institut für Biologie II, Abt. Zoologie und Humanbiologie, Worringerweg 3, 52056 Aachen, Germany

**Keywords:** *Drosophila melanogaster*, Follicle cell, Epithelium, Bioelectricity, Intracellular pH, Membrane potential, Cell polarity, Pattern formation, Ion pump, Ion channel

## Abstract

**Background:**

Alterations of bioelectrical properties of cells and tissues are known to function as wide-ranging signals during development, regeneration and wound-healing in several species. The *Drosophila* follicle-cell epithelium provides an appropriate model system for studying the potential role of electrochemical signals, like intracellular pH (pH_i_) and membrane potential (V_mem_), during development. Therefore, we analysed stage-specific gradients of pH_i_ and V_mem_ as well as their dependence on specific ion-transport mechanisms.

**Results:**

Using fluorescent indicators, we found distinct alterations of pH_i_- and V_mem_-patterns during stages 8 to 12 of oogenesis. To determine the roles of relevant ion-transport mechanisms in regulating pH_i_ and V_mem_ and in establishing stage-specific antero-posterior and dorso-ventral gradients, we used inhibitors of Na^+^/H^+^-exchangers and Na^+^-channels (amiloride), V-ATPases (bafilomycin), ATP-sensitive K^+^-channels (glibenclamide), voltage-dependent L-type Ca^2+^-channels (verapamil), Cl^−^-channels (9-anthroic acid) and Na^+^/K^+^/2Cl^−^-cotransporters (furosemide). Either pH_i_ or V_mem_ or both parameters were affected by each tested inhibitor. While the inhibition of Na^+^/H^+^-exchangers (NHE) and amiloride-sensitive Na^+^-channels or of V-ATPases resulted in relative acidification, inhibiting the other ion-transport mechanisms led to relative alkalisation. The most prominent effects on pH_i_ were obtained by inhibiting Na^+^/K^+^/2Cl^−^-cotransporters or ATP-sensitive K^+^-channels. V_mem_ was most efficiently hyperpolarised by inhibiting voltage-dependent L-type Ca^2+^-channels or ATP-sensitive K^+^-channels, whereas the impact of the other ion-transport mechanisms was smaller. In case of very prominent effects of inhibitors on pH_i_ and/or V_mem_, we also found strong influences on the antero-posterior and dorso-ventral pH_i_- and/or V_mem_-gradients. For example, inhibiting ATP-sensitive K^+^-channels strongly enhanced both pH_i_-gradients (increasing alkalisation) and reduced both V_mem_-gradients (increasing hyperpolarisation). Similarly, inhibiting Na^+^/K^+^/2Cl^−^-cotransporters strongly enhanced both pH_i_-gradients and reduced the antero-posterior V_mem_-gradient. To minor extents, both pH_i_-gradients were enhanced and both V_mem_-gradients were reduced by inhibiting voltage-dependent L-type Ca^2+^-channels, whereas only both pH_i_-gradients were reduced (increasing acidification) by inhibiting V-ATPases or NHE and Na^+^-channels.

**Conclusions:**

Our data show that in the *Drosophila* follicle-cell epithelium stage-specific pH_i_- and V_mem_-gradients develop which result from the activity of several ion-transport mechanisms. These gradients are supposed to represent important bioelectrical cues during oogenesis, e.g., by serving as electrochemical prepatterns in modifying cell polarity and cytoskeletal organisation.

**Electronic supplementary material:**

The online version of this article (10.1186/s12861-019-0192-x) contains supplementary material, which is available to authorized users.

## Background

The development and maintenance of complex multicellular structures, like tissues and organs, is controlled by an interplay of various regulatory processes. Besides genetical and biochemical mechanisms, bioelectrical phenomena, i.e. localised ion fluxes, gradients of ion concentrations, intracellular pH (pH_i_) and membrane potential (V_mem_), are known to function as wide-ranging signals to guide polarity in development, regeneration and wound-healing [1–5]. It has been shown that gradual modifications of bioelectrical properties mediate cellular processes like migration [6], proliferation [7], differentiation [8–10], and cell-cycle control [11, 12] in various species. For example, V_mem_ plays a role in specifying the left-right axis in *Xenopus* and chick embryos [13], in the regeneration of either head or tail in planarians [14], in zebrafish pigment-pattern formation [15], and in planar cell-polarity pathway regulation in *Drosophila* epithelial tissues [16]. Defects in ion transport have been associated with several human diseases, like e.g. cancer development or Alzheimer’s disease, or with developmental defects caused by human channelopathies [17–21].

The generation and maintenance of electrochemical gradients within cells or tissues requires a polarised distribution and/or activation of specific ion-transport mechanisms [22, 23]. In addition, gap junctions are a prerequisite in coupling groups of cells electrically [24–26]. In contrast to classical methods, like e.g., single-cell electrode measurements, new techniques using specific fluorescent probes allow visualisation and analysis of the spatiotemporal characteristics of pH_i_ and V_mem_ in a large number of cells and in whole tissues [27, 28].

The follicle-cell epithelium (FCE) of the *Drosophila* ovary provides an appropriate model system for studying bioelectrical phenomena during epithelial development [29–33]. It has been shown that in *Drosophila* ovarian follicles stage-specific patterns of extracellular currents [29, 30], V_mem_ [31, 32, 34], and pH_i_ [32] exist that depend mainly on the exchange of protons, potassium ions and sodium ions [31, 34–36]. For studying and manipulating pH_i_ and V_mem_ in the FCE, we used the fluorescent pH-indicator 5-CFDA,AM and the potentiometric dye DiBAC_4_(3) as well as several inhibitors of ion-transport mechanisms.

**Fig. 1:**
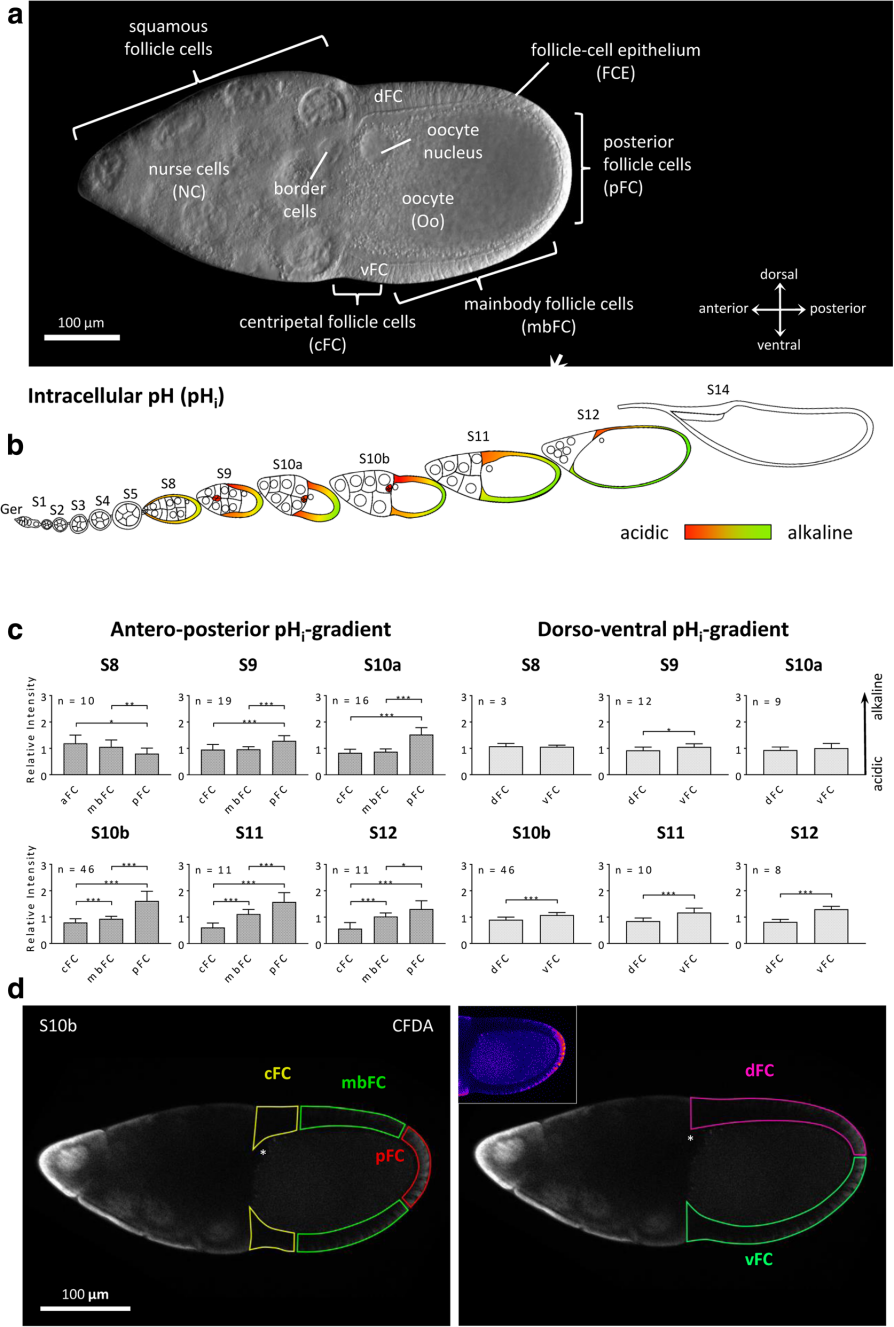
Development of pH_i_-gradients in the FCE during S8–12 (CFDA; SIM). **a** Types of germ-line and somatic cells of the *Drosophila* follicle. At stage 10b (S10b), the oocyte (Oo, posterior) constitutes almost one half of the follicle’s volume. The anterior half is formed by 15 nurse cells (NC). The 16 germ-line cells (Oo and NC) are covered by a single-layered somatic follicle-cell epithelium (FCE). The columnar FCE surrounding the oocyte is subdivided into three follicle-cell (FC) populations: centripetally migrating FC (cFC), mainbody FC (mbFC) and posterior FC (pFC). The dorsal FCE (dFC) is thicker than the ventral FCE (vFC). Squamous FC surround the NC population. The oocyte nucleus and the border cells, a cluster of migratory follicle cells, lie close to each other near the dorsal side (DIC image). **b** Schematic drawing of an ovariole showing pH_i_ in the analysed stages S8–12 (cf. Additional file 1: Figure S1 for examples; Ger, germarium). **c** In S8, anterior FC (aFC) are more alkaline compared to pFC. During S9–12, an antero-posterior (a-p) gradient establishes with relatively acidic cFC and relatively alkaline pFC. From S9 and, in particular, from S10b onwards, a dorso-ventral (d-v) gradient with relatively alkaline vFC and relatively acidic dFC develops. To analyse the a-p and d-v gradients, the fluorescence intensities of the different FC types (3 < *n* < 46 follicles per stage) were measured and normalised using the fluorescence intensity in the whole FCE of the respective follicle (*relative intensity*). Mean values, shown with their standard deviation (cf. Additional file 2: Table S1), were compared using an unpaired t-test (* *p* < 0.05; ** *p* < 0.01; *** *p* < 0.001). **d** Optical median sections of a CFDA-stained S10b-follicle. Inset shows a pseudocolour image of the same section. Low fluorescence intensity (blue) indicates lower pH_i_ (relatively acidic) while high fluorescence intensity (pink) indicates higher pH_i_ (relatively alkaline). Asterisk, oocyte nucleus (dorsal)

The *Drosophila* follicle consists of 16 germ-line cells, 15 nurse cells (NC) and one oocyte (Oo), surrounded by a single-layered somatic FCE [37]. During the course of oogenesis, the FCE differentiates into several morphologically distinct follicle-cell (FC) populations (Fig. 1a) [38–40]. In addition to establishing embryonic polarity [41] and building up the eggshell [40], the FCE plays a significant role in shaping the elongated egg [42, 43], a process which requires planar cell polarity and a polarised arrangement of the cytoskeleton.

The purpose of the present study is to clarify the roles that various ion-transport mechanisms play in regulating pH_i_ and V_mem_ and in generating stage-specific electrochemical gradients in the FCE. Such gradients are likely to be involved in regulating, e.g., cell polarity, cell migration, and the organisation of the cytoskeleton during oogenesis of *Drosophila*.

## Results

### Stage-specific pH_i_-patterns

**Fig. 2:**
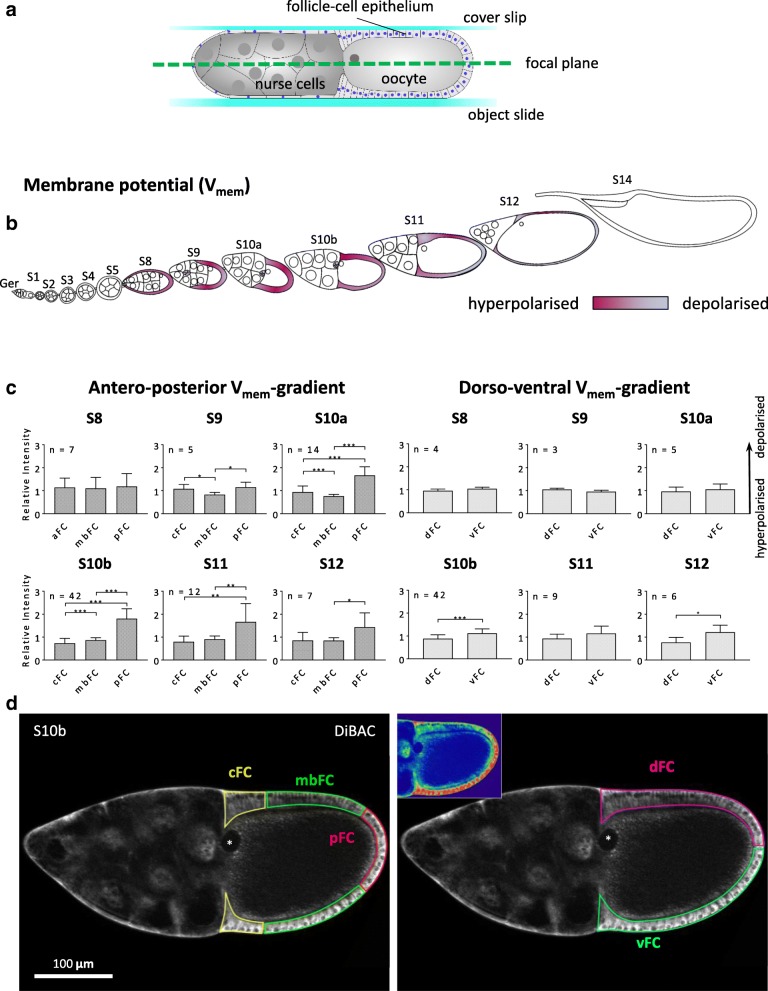
Development of V_mem_-gradients in the FCE during S8–12 (DiBAC; SIM). **a** Schematic drawing of a S10b-follicle placed between an object slide and a cover slip. The focal plane of median optical sections generated by the ApoTome (SIM) is shown as dashed green line. **b** Schematic drawing of an ovariole showing V_mem_ in the analysed stages S8–12 (cf. Additional file 1: Figure S2 for examples). **c** During S9–12, an a-p gradient establishes with relatively hyperpolarised cFC and relatively depolarised pFC. From S10b onwards, a d-v gradient with relatively depolarised vFC and relatively hyperpolarised dFC develops (cf. Additional file 2: Table S2). For abbreviations and statistics, see legend to Fig. 1. **d** Optical median sections of a DiBAC-stained S10b-follicle. Inset shows a pseudocolour image of the same section. Low fluorescence intensity (green) indicates relative hyperpolarisation while high fluorescence intensity (red) indicates relative depolarisation

We analysed the pH_i_ in the FCE during the course of oogenesis. In vitellogenic stages (S8–12), we found distinct alterations of the pH_i_-patterns (Figs. 1b-d and 2a; for typical follicles, see Additional file 1: Figure S1; for variability between follicles of the same stage, see Additional file 2: Table S1).

#### Antero-posterior gradient

In S8, the pH_i_ in the FCE is relatively acidic compared to the pH_i_ in the germ-line cells. An antero-posterior (a-p) gradient is visible, with more alkaline pH_i_ in the anterior FC (aFC) and more acidic pH_i_ in the posterior FC (pFC). In S9 and 10a, the a-p gradient reverses and the centripetal FC (cFC) show more acidic pH_i_ than the pFC. At S10b, this a-p gradient becomes very distinct and is preserved during S11 and 12. In contrast to earlier stages, the pH_i_ in the FCE is now more alkaline compared to the pH_i_ in the germ-line cells.

#### Dorso-ventral gradient

In S8, the FCE does not yet exhibit a dorso-ventral (d-v) gradient. A significant gradient with relatively acidic dorsal and relatively alkaline ventral FC arises in S9 and increases during S10b-12.

### Stage-specific V_mem_-patterns

During the course of vitellogenesis (S8–12), we also observed distinct changes in the V_mem_-patterns (Fig. 2; for typical follicles, see Additional file 1: Figure S2; for variability between follicles of the same stage, see Additional file 2: Table S2).

#### Antero-posterior gradient

Up to S8, all FC show a similar V_mem_ which is relatively depolarised compared to the germ-line cells. At S9 and 10a, an a-p gradient starts to establish with relatively depolarised FC in the centripetal and posterior regions of the follicle (cFC and pFC) compared to the mainbody FC (mbFC). During S10b-12, the gradient changes so that only the pFC are depolarised compared to mbFC and cFC.

#### Dorso-ventral gradient

During S8-10a, a significant d-v gradient could not be detected in the FCE. However, a d-v gradient emerges during S10b-12, with relative depolarisation on the ventral side of the follicle (vFC). Dorsal cFC show a striking increase in depolarisation during late S10b and 11. But on average, the dorsal FCE is hyperpolarised compared to the ventral FCE.

### Inhibition of ion-transport mechanisms

Six inhibitors were used to determine the roles that specific ion-transport mechanisms play in the regulation of pH_i_ and V_mem_ as well as in the generation of electrochemical gradients in the FCE during S10b. We found that either pH_i_ or V_mem_ or both parameters were affected by each tested inhibitor.

### Effects of inhibitors on pH_i_

#### WFM-experiment

**Fig. 3:**
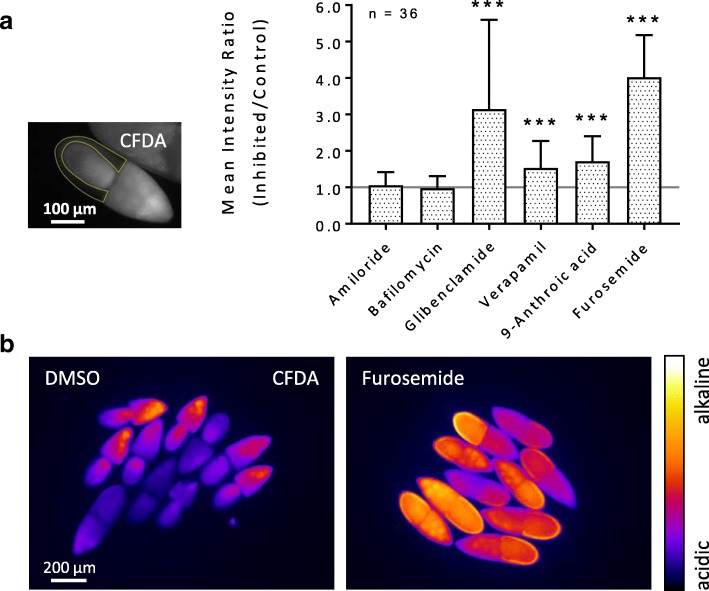
Inhibitors of ion-transport mechanisms exert influence on the pH_i_ in the FCE during S10b (WFM-experiment; CFDA). **a** WFM-fluorescence images were used to measure the fluorescence intensity in the columnar FCE (marked yellow) of every single follicle. While glibenclamide (ATP-sensitive K^+^-channels) and furosemide (Na^+^/K^+^/2Cl^−^-cotransporters) led to strong alkalisation, the alkalising effects of verapamil (L-type Ca^**2**+^-channels) and 9-anthroic acid (Cl^−^-channels) were smaller. Amiloride (NHEs and Na^+^-channels) and bafilomycin (V-ATPases) showed no significant effects. To analyse and compare the effects of the inhibitors, averaged values (of 12 time points during 60 min of inhibition) of three experiments per inhibitor were summed up and normalised (*mean intensity ratio*). Mean values, shown with their standard deviation (cf. Additional file 2: Table S3), were compared using an unpaired t-test (* p < 0.05; ** p < 0.01; *** p < 0.001). **b** Pseudocolour fluorescence images after 60 min of incubation. Furosemide led to strong alkalisation of the whole follicle. In contrast to the control (DMSO), the columnar FCE exhibits an even stronger fluorescence intensity than the germ-line cells

The inhibitors furosemide (Na^+^/K^+^/2Cl^−^-cotransporters), glibenclamide (ATP-sensitive K^+^-channels), 9-anthroic acid (Cl^−^-channels) and verapamil (L-type Ca^2+^-channels) showed significant alkalising effects in the FCE. The inhibitors amiloride (NHEs and Na^+^-channels) and bafilomycin (V-ATPases) had no significant effects on the pH_i_ in the FCE under these experimental conditions. The strongest effects were observed with both glibenclamide and furosemide (Fig. 3, Additional file 2: Table S3).

#### SIM-experiment

**Fig. 4:**
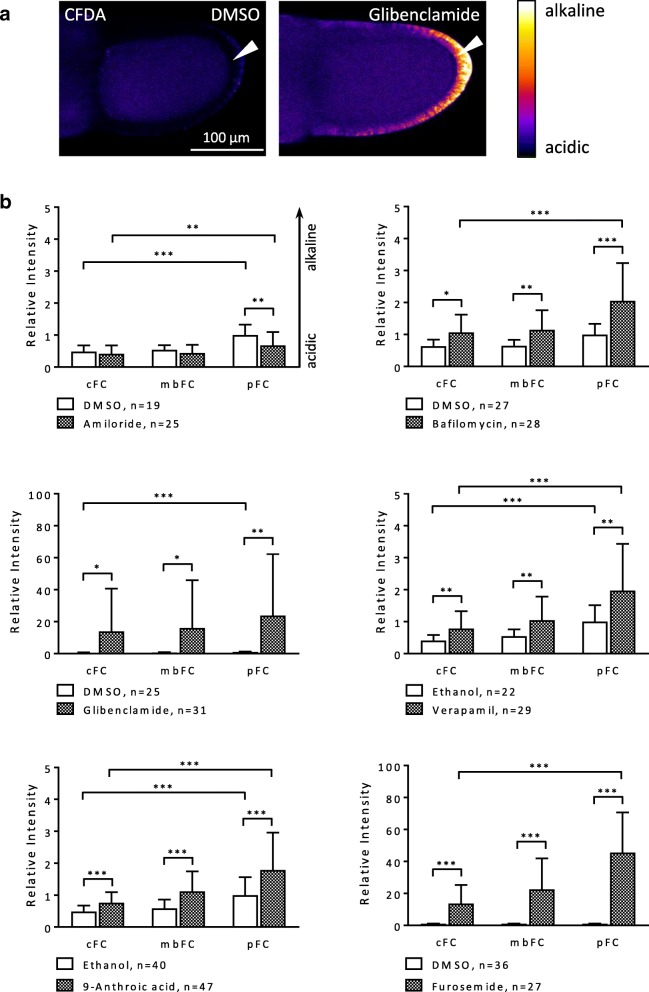
The a-p pH_i_-gradient in the FCE is affected by all inhibitors during S10b (SIM-experiment; CFDA). **a** Pseudocolour SIM-fluorescence images of S10b-follicles. Compared to the control (DMSO), blocking with glibenclamide for 20 min resulted in strong alkalisation of the FCE which was most prominent in pFC (arrowhead). **b** Especially glibenclamide and furosemide, but also verapamil and 9-anthroic acid led to alkalisation in all FC types. Glibenclamide and furosemide resulted in a considerably steeper a-p gradient (enlargement of the angle × 20 and × 150, respectively). Verapamil and 9-anthroic acid enhanced the a-p gradient as well, but to a lesser extent (both × 2). The increase in fluorescence intensity as well as in the inclination of the a-p gradient (× 2) caused by bafilomycin was due to the alkalisation of vesicles (see Fig. 5 b) and, therefore, to the acidification of the cytoplasm resulting in a shallower a-p gradient. Amiloride led to acidification of pFC, thus slightly reducing the angle of the a-p gradient (× 0.5) as well. For each inhibitor, at least five repetitions of the experiment were performed. Normalised values of the single experiments were merged into one evaluation (*relative intensity*). Mean values, shown with their standard deviation (cf. Additional file 2: Table S5), were compared using an unpaired t-test (* *p* < 0.05; **p < 0.01; *** *p* < 0.001)

**Fig. 5:**
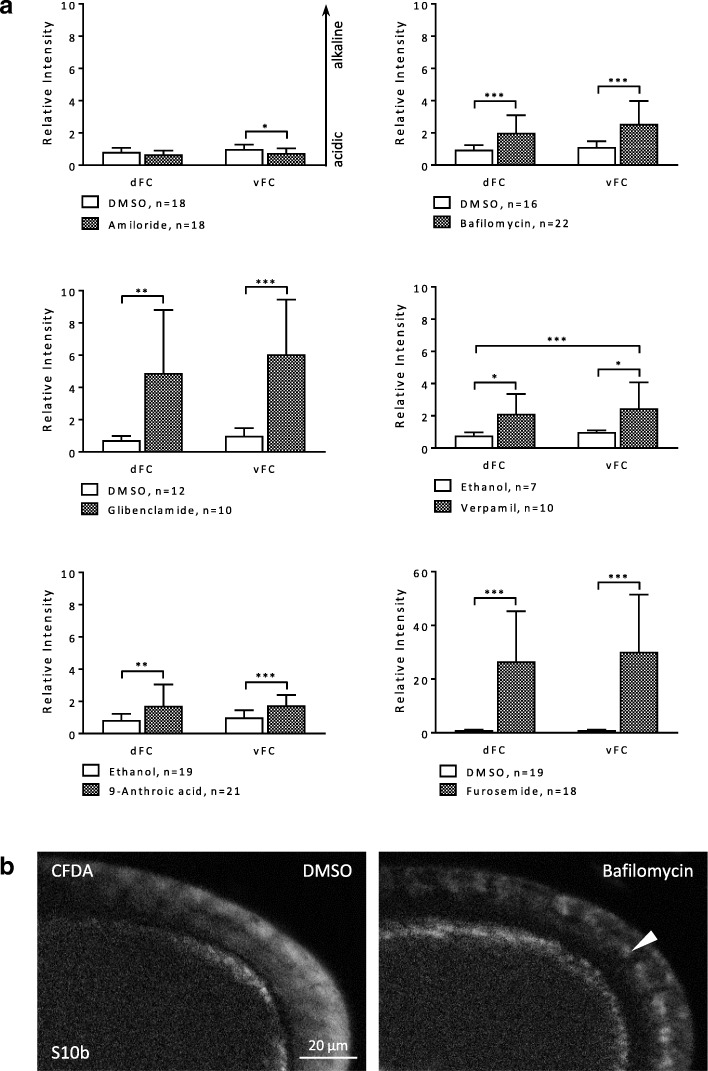
**a** All inhibitors exert influence on the d-v pH_i_-gradient in the FCE during S10b (SIM-experiment; CFDA). Especially glibenclamide and furosemide, but also verapamil and 9-anthroic acid led to alkalisation in both FC types. Verapamil, glibenclamide, and furosemide resulted in a steeper d-v gradient (angle × 2, × 5, and × 100, respectively). The increase in fluorescence intensity as well as in the inclination of the d-v gradient (× 3) caused by bafilomycin was due to the alkalisation of vesicles (see **b**), leading to increasing cytoplasmic acidification as well as to a shallower d-v gradient. Amiloride and 9-anthroic acid led to a stronger acidification of vFC, thus reducing the angle of the d-v gradient (× 0.5 and × 0.1, respectively) as well (cf. Additional file 2: Table S5). For statistics, see legend to Fig. 4. **b** Alkalisation of vesicles in the FCE by bafilomycin. SIM-fluorescence images of S10b-follicles incubated in R-14 medium with bafilomycin or with DMSO (control) for 20 min. Inhibition of V-ATPases led to a more granular staining (arrowhead) compared to the controls, indicating alkalisation of vesicles. In addition, the cytoplasm of the FC appeared darker than in the controls, indicating cytoplasmic acidification

The treatment with the inhibitors furosemide, glibenclamide, 9-anthroic acid and verapamil, respectively, resulted again in alkalisation. These effects were significant in all types of FC and particularly distinct with furosemide and glibenclamide (Figs. 4 and 5a, Additional file 2: Table S5). Amiloride led to slight acidification which was significant in pFC. The increase in fluorescence intensity elicited by bafilomycin was due to granular staining in the FCE (Fig. 5 b). Considering the localisation of V-ATPases in plasma membranes and in vesicle membranes of FC [33, 46], it is obvious that bafilomycin caused alkalisation of vesicles, while the cytoplasm of FC became more acidic. Modifications of the a-p and/or d-v pH_i_-gradients were observed with all tested inhibitors. The influences were particularly strong with both glibenclamide and furosemide, which enhanced the a-p and d-v gradients (increasing angle; alkalisation), as did verapamil. 9-Anthroic acid resulted in a shallower d-v gradient (decreasing angle; acidification) because of its stronger effect on vFC, whereas the a-p gradient became slightly enhanced (alkalisation). Amiloride, on the other hand, led to both shallower a-p and d-v gradients (acidification), as did bafilomycin (increasing cytoplasmic acidification due to increasing vesicle alkalisation). The inclinations of the gradients were inferred from the mean values of relative fluorescence intensity depicted in Figs. 4b and 5a.

### Effects of inhibitors on V_mem_

#### WFM-experiment

**Fig. 6:**
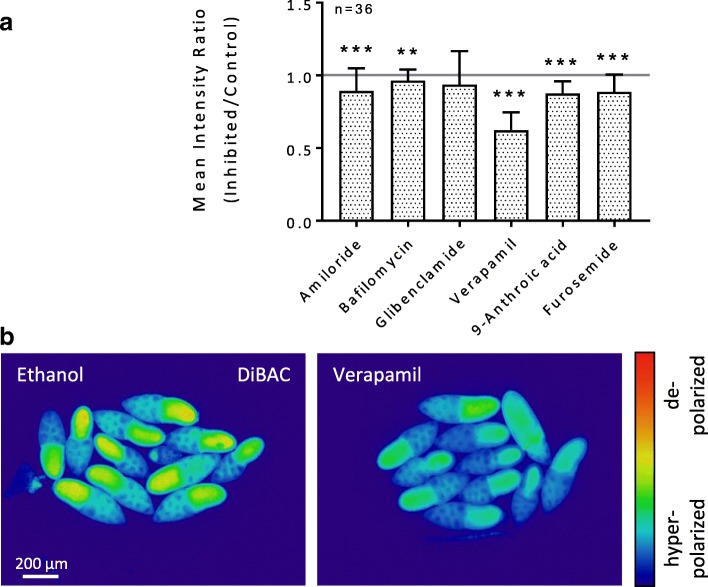
Inhibitors of ion-transport mechanisms exert influence on the V_mem_ in the FCE during S10b (WFM-experiment; DiBAC). **a** WFM-fluorescence images were used to measure the fluorescence intensity in the columnar FCE of every single follicle. The measured area was as shown in Fig. 3 a. While verapamil led to strong hyperpolarisation, amiloride, bafilomycin, 9-anthroic acid, and furosemide led to slight hyperpolarisation. The hyperpolarising effect of glibenclamide was not significant (cf. Additional file 2: Table S4). For abbreviations and statistics, see legend to Fig. 3. **b** Pseudocolour fluorescence images after 60 min of incubation. Verapamil led to a strong decrease in fluorescence intensity (hyperpolarisation) in the whole follicle

Almost all tested inhibitors (verapamil, amiloride, 9-anthroic acid, furosemide and bafilomycin) had significant hyperpolarising effects on the V_mem_ in the FCE. The strongest effect was observed with verapamil, the weakest with bafilomycin. Glibenclamide caused a slight hyperpolarisation which was not significant. Depolarisation was not observed (Fig. 6, Additional file 2: Table S4).

#### SIM-experiment

**Fig. 7:**
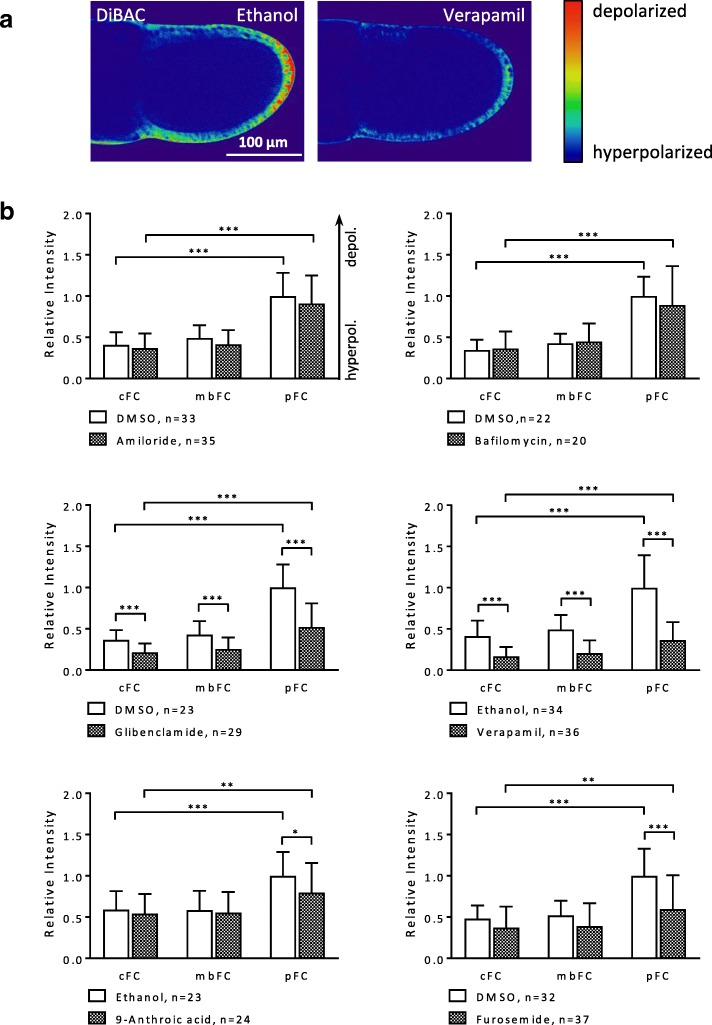
The a-p V_mem_-gradient in the FCE is affected by most inhibitors during S10b (SIM-experiment; DiBAC). **a** Pseudocolour SIM-fluorescence images of S10b-follicles. Compared to the control (ethanol), blocking with verapamil for 20 min resulted in strong hyperpolarisation of the FCE. **b** Glibenclamide and verapamil led to strong hyperpolarisation in all FC types, but especially in pFC, thus lowering the inclination of the a-p gradient (angle × 0.5 and × 0.3, respectively). Furosemide and 9-anthroic acid hyperpolarised only the pFC, thereby lowering the a-p gradient as well (both × 0.5). Amiloride and bafilomycin showed no significant effects on the a-p gradient (cf. Additional file 2: Table S6). For statistics, see legend to Fig. 4

**Fig. 8:**
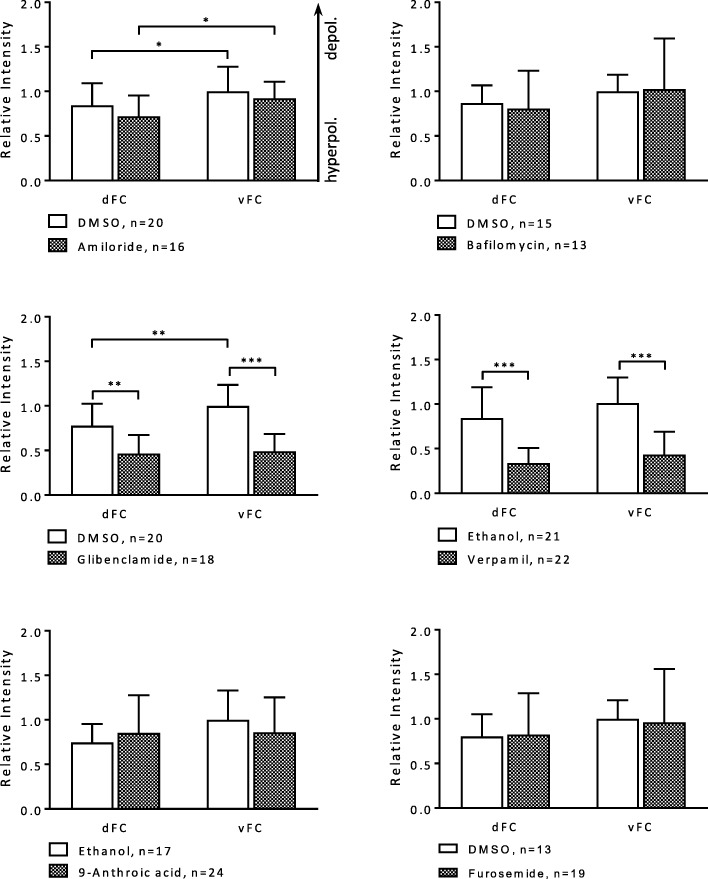
Some inhibitors exert influence on the d-v V_mem_-gradient in the FCE during S10b (SIM-experiment; DiBAC). Glibenclamide and verapamil led to hyperpolarisation in both FC types, but especially in vFC, thus lowering the inclination of the d-v gradient (angle × 0.1 and × 0.5, respectively). Amiloride, bafilomycin, 9-anthroic acid and furosemide showed no significant effects on the d-v gradient (cf. Additional file 2: Table S6). For statistics, see legend to Fig. 4

All inhibitors caused more or less hyperpolarisation in the FCE (Figs. 7 and 8, Additional file 2: Table S6). The strongest effects on V_mem_ were observed with verapamil and glibenclamide, the weakest with amiloride and bafilomycin (not significant). Glibenclamide and verapamil lowered the a-p gradient as well as the d-v gradient (decreasing angle; hyperpolarisation), whereas furosemide and 9-anthroic acid had prominent decreasing effects only on the a-p gradient. The inclinations of the gradients were inferred from the mean values of relative fluorescence intensity depicted in Figs. 7b and 8.

## Discussion

### Stage-specific pH_i_- and V_mem_-gradients are established in the FCE

We have shown that the FCE exhibits stage-specific a-p and d-v gradients of pH_i_ and V_mem_, respectively, which are most distinctive during S10b. The pH_i_- and V_mem_-gradients arise approximately at the same stage, but they are not congruent. The pH_i_ increases (alkalisation) from anterior (cFC) to posterior (pFC) and from dorsal (dFC) to ventral (vFC), and, therefore, the dorsal cFC possess the most acidic pH_i_.

In a-p direction, the developing V_mem_-gradient differs from the pH_i_-gradient. Since the mbFC are relatively hyperpolarised compared to the cFC and pFC, no continuous a-p V_mem_-gradient exists. However, from dFC to vFC, the V_mem_ becomes continuously depolarised. For both pH_i_ and V_mem_, the stages differ during which the a-p and d-v gradients become established. Since they start in S9, the a-p gradients are already existing when the d-v gradients become obvious during S10b.

It is expected that the establishment of electrochemical gradients is depending on the asymmetric distribution and/or activity of ion-transport mechanisms in different types of FC. Recent studies have shown that e.g. gap junctions, V-ATPases, Na^+^/K^+^-pumps and Na^+^-channels are enriched in certain regions of the FCE compared to other regions [32, 33, 36, 44–46]. To function as temporal and spatial signals during development, electrochemical gradients and/or local changes of pH_i_ and V_mem_ have to be translated into cellular responses. For example, this could be achieved by voltage-dependent L-type Ca^2+^-channels that become asymmetrically activated in the FCE [32].

### Inhibitors of ion-transport mechanisms affect pH_i_ and V_mem_ in the FCE

**Fig. 9:**
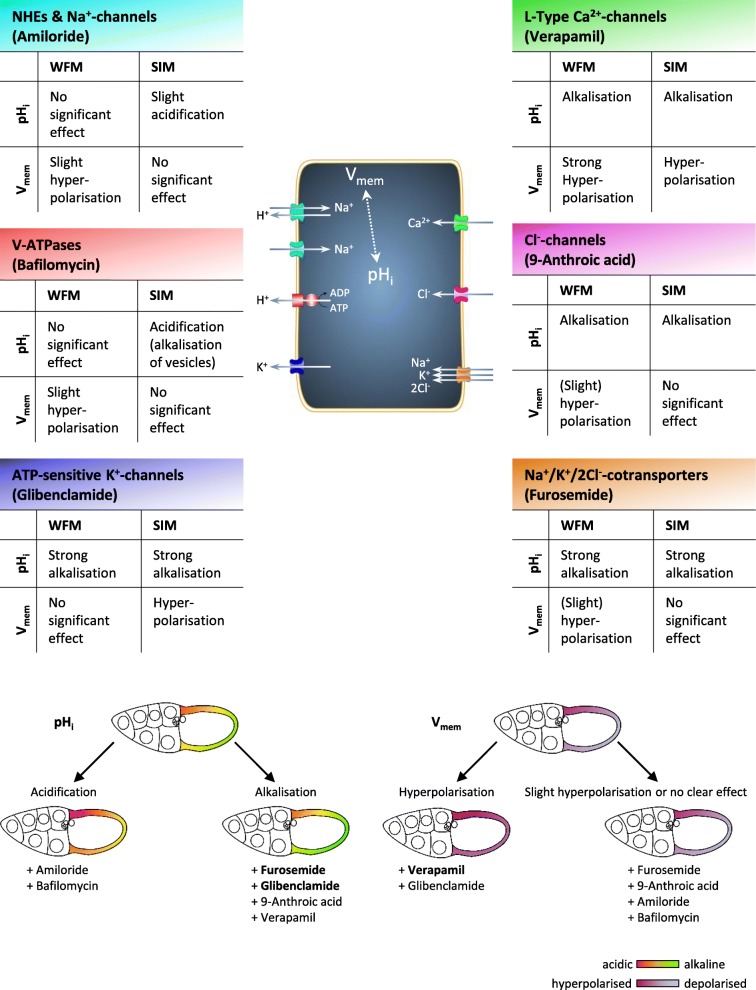
Summary of the effects of inhibitors on pH_i_ and V_mem_ in the WFM- and SIM-experiments. Although both experimental conditions led to somewhat different results (upper part), the effects of the inhibitors can be reliably determined (lower part). While inhibition of both Na^+^/K^+^/2Cl^−^-cotransporters (furosemide) and ATP-sensitive K^+^-channels (glibenclamide) resulted in the most prominent effects on pH_i_ (strong alkalisation), inhibition of voltage-dependent L-type Ca^2+^-channels (verapamil) led to the most prominent effect on V_mem_ (strong hyperpolarisation)

To identify mechanism that are involved in regulating pH_i_ and V_mem_ in the FCE, we used inhibitors of several ion-transport mechanisms that have already been detected and/or localised in the *Drosophila* ovary [32, 33, 44, 47–50]. All used inhibitors affected either pH_i_ or V_mem_ or both parameters in S10b (for summary, see Fig. 9). If an inhibitory effect was detectable, it could be observed throughout the entire columnar FCE - though, in different FC types, often to varying extents.

The most prominent effects on pH_i_ were obtained with both glibenclamide (ATP-sensitive K^+^-channels) and furosemide (Na^+^/K^+^/2Cl^−^-cotransporters). These inhibitors led to strong relative alkalisation in all FC types by blocking H^+^-transport indirectly. Inhibition of ATP-sensitive K^+^-channels by glibenclamide reduces K^+^-efflux. Since the intracellular K^+^-concentration and the pH_i_ are known to be interrelated, a higher intracellular K^+^-concentration would reduce H^+^-influx (K^+^/H^+^-antiport [51, 52]). In addition, it has been shown that K^+^-uptake into *Drosophila* follicles is strongly dependent on extracellular pH [36]. Na^+^/K^+^/2Cl^−^-cotransport, which can be blocked by furosemide (or bumetanide, an analogue of furosemide), is typically coupled with Cl^−^/HCO_3_^−^-antiport, so that furosemide has no impact on Cl^−^-transport in total [53, 54]. Due to this coupling, HCO_3_^−^-transport might be affected, causing a change of pH_i_ in the FCE [55, 56]. The alkalisation obtained with 9-anthroic acid, which blocks Cl^−^-channels, is also likely to be due to an effect on Cl^−^/HCO_3_^−^-antiport.

Amiloride (NHEs and Na^+^-channels) and bafilomycin (V-ATPases), that are both direct inhibitors of H^+^-transport, led to slight acidification of the FCE. While NHEs are known for their role in pH_i_-homeostasis [47, 56–58], one of the key functions of V-ATPases, besides acidification of cytoplasmic vesicles, is to energise the plasma membranes of most insect cells [59, 60]. Since the generated H^+^-gradient drives secondary active transport mechanisms like NHEs [51, 60], a strong effect of bafilomycin on pH_i_ was not expected. Similarly, the relatively small impact of amiloride on pH_i_ is supposed to be due to compensatory effects exerted by other ion transporters.

V_mem_ in the FCE was clearly influenced by glibenclamide (ATP-sensitive K^+^-channels) and, most strongly, by verapamil (voltage-dependent L-type Ca^2+^-channels). Although Ca^2+^-channels are not likely to contribute much to V_mem_ directly, blocking of Ca^2+^-influx can result in diverse cellular reactions, since Ca^2+^ is a second messenger [61]. A strong effect of amiloride on V_mem_ was not expected, since Na^+^/H^+^-antiport is electroneutral. Moreover, the effect of blocking Na^+^-channels is relatively weak and can be compensated by other ion-transport mechanisms. Compensatory effects always have to be taken into account, and they are supposed to be the reason for the absence of a strong impact of bafilomycin (V-ATPases) on V_mem_ as well. Similarly, inhibition with furosemide (Na^+^/K^+^/2Cl^−^-cotransporters) and 9-anthroic (Cl^−^-channels) had only minor effects on V_mem_.

### Inhibitors of ion-transport mechanisms affect pH_i_- and V_mem_-gradients in the FCE

**Fig. 10:**
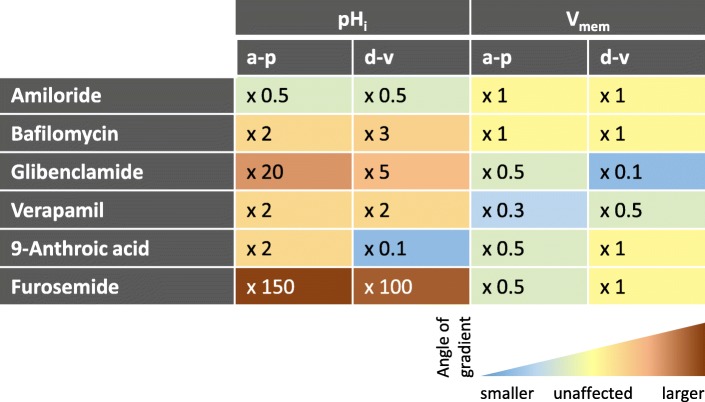
Summary of the influences of inhibitors on the inclinations of the a-p and/or d-v pH_i_- and V_mem_-gradients. Blocking of ion-transport mechanisms resulted in very different effects on the various gradients of relative fluorescence intensity. While, e.g. for the a-p pH_i_-gradient, × 0.5 represents a reduction of the angle (decreasing alkalisation, i.e. increasing acidification) by 50% (amiloride; NHEs and Na^+^-channels), × 150 means a 150fold enlargement (furosemide; Na^+^/K^+^/2Cl^−^-cotransporters) of the angle (increasing alkalisation). The enlargement of the angles caused by bafilomycin (V-ATPases) was due to the alkalisation of vesicles resulting in increased cytoplasmic acidification as well as in shallower pH_i_-gradients. E.g., for the d-v V_mem_-gradient, × 0.1 represents a reduction of the angle (decreasing depolarisation, i.e. increasing hyperpolarisation) by 90% (glibenclamide; ATP-sensitive K^+^-channels). The inclinations of the gradients were inferred from the mean values of relative fluorescence intensity shown in Figs. 4b and 5a (pH_i_-gradients) and in Figs. 7b and 8 (V_mem_-gradients), respectively

Since with some inhibitors different types of FC were affected to different extents, the a-p and/or d-v gradients during S10b became modified in several ways (for summary, see Fig. 10).

The treatment with glibenclamide (ATP-sensitive K^+^-channels) resulted in both an enhancement of the a-p and d-v pH_i_-gradients (increasing alkalisation) and a reduction of the a-p and d-v V_mem_-gradients (increasing hyperpolarisation). This could be due to the higher concentrations of ATP-sensitive K^+^-channels found in dorsal cFC (unpublished results). It has been shown that both acidic pH and low K^+^-concentrations result in reduced gap-junctional communication in *Drosophila* follicles [48]. Therefore, glibenclamide is supposed to enhance gap-junctional communication via higher intracellular K^+^-concentrations as well as alkalisation of pH_i_, leading to increasing hyperpolarisation in the V_mem_-gradients. Furosemide (Na^+^/K^+^/2Cl^−^-cotransporters) also led to markedly steeper a-p and d-v pH_i_-gradients as well as to a shallower a-p V_mem_-gradient. Whether these effects depend on an unequal distribution of Na^+^/K^+^/2Cl^−^-cotransporters remains to be analysed. Likewise, the distributions of NHEs (amiloride) as well as Cl^−^-channels (9-anthroic acid), which both modified electrochemical gradients in the FCE, are not yet known. However, it has been shown that V-ATPases are accumulated in the membranes of pFC and vFC [32, 33]. A stronger effect of bafilomycin on these FC types led to reduction of a-p and d-v pH_i_-gradients (increasing cytoplasmic acidification due to increasing vesicle alkalisation). The application of verapamil both enhanced the a-p and d-v pH_i_-gradients and reduced the a-p and d-v V_mem_-gradients (increasing hyperpolarisation). This is in accordance with the observed higher concentrations of activated L-type Ca^2+^-channels in pFC and vFC [32]. It is known that high intracellular Ca^2+^-concentrations result in reduced gap-junctional communication in *Drosophila* follicles [46]. Therefore, verapamil is supposed to enhance gap-junctional communication by lowering intracellular Ca^2+^-concentrations, leading to both increasing alkalisation in pH_i_-gradients and increasing hyperpolarisation in V_mem_-gradients.

## Conclusion

In the FCE of *Drosophila*, gap junctions and ion-transport mechanisms, like NHEs, Na^+^-channels, V-ATPases, ATP-sensitive K^+^-channels, voltage-dependent L-type Ca^2+^-channels, Cl^−^-channels and Na^+^/K^+^/2Cl^−^-cotransporters, are involved in the generation of stage-specific pH_i_- and V_mem_-gradients. Since bioelectrical phenomena are known to affect, e.g., cytoskeletal dynamics, such gradients are supposed to serve as electrochemical prepatterns that control planar cell polarity and guide gradual changes in cytoskeletal organisation. In the FCE, a particular arrangement of cytoskeletal elements is involved in shaping the follicle [42, 43, 62], and stage-specific correlations with pH_i_- and V_mem_-gradients can be observed (unpublished results). Therefore, manipulating the electrochemical gradients and analysing related changes in microfilament and microtubule patterns will help to understand the influence of bioelectrical cues during development (I. Weiß and J. Bohrmann, manuscript in prep.).

## Methods

### Preparation of *Drosophila* follicles

Oregon R (wild-type) *Drosophila melanogaster* were reared at 20–23 °C on standard medium with additional fresh yeast. 2–3 days old females were killed by crushing the head and thorax with tweezers without anaesthesia. The ovaries were dissected and single follicles of all stages (S1–14) were isolated. The preparations were carried out in R-14 medium [63] which is best suited for in-vitro culture of *Drosophila* follicles [64].

### Analysis of intracellular pH

Intracellular pH (pH_i_) of FC was analysed using the pH-sensitive fluorescent dye CFDA (5-carboxyfluorescein diacetate, acetoxymethyl ester; 5-CFDA,AM; Molecular Probes/Thermo Fisher Scientific, USA) [27, 32]. Relative fluorescence intensities were stated, i. e. lower fluorescence intensity indicates lower pH_i_ (more acidic) while higher fluorescence intensity indicates higher pH_i_ (more alkaline). Living follicles were incubated for 20 or 60 min, depending on the type of experiment (see below), in R-14 medium containing 4 μM CFDA (dissolved in dimethyl sulfoxide; DMSO). For controls, see Additional file 1: Fig. S3.

### Analysis of membrane potential

To analyse membrane potentials (V_mem_) of FC, the voltage-sensitive fluorescent dye DiBAC (bis-(1,3-dibutylbarbituric acid) trimethine oxonol; DiBAC_4_(3); Molecular Probes) was used [28, 32]. Relative fluorescence intensities were stated, i. e. lower fluorescence intensity indicates relative hyperpolarisation and higher fluorescence intensity indicates relative depolarisation. Living follicles were incubated for 20 or 60 min, depending on the type of experiment (see below), in R-14 medium containing 4 μM DiBAC (dissolved in 70% ethanol).

### Fluorescence microscopy and optical sectioning

To investigate pH_i_ and V_mem_ as well as their changes in detail, two types of experiments were performed. ImageJ (NIH, USA) was used to generate pseudocolour images.

#### WFM-experiment

Groups of four to ten follicles were imaged for 60 min during staining in covered glass block dishes on a Zeiss Axiovert 200 wide-field fluorescence microscope (WFM), equipped with a Hamamatsu Orca ER camera, using a × 5 objective and a × 1.6 optovar. An image was taken every 5 min. Settings and exposure time remained unchanged.

#### SIM-experiment

Single follicles were imaged in R-14 medium after staining for 20 min on a Zeiss AxioImager.M2 structured-illumination microscope (SIM), equipped with a Zeiss ApoTome and a Zeiss AxioCamMRm camera, using a × 20 objective. Median optical sections were produced as shown in Fig. 2 a.

### Inhibition of ion-transport mechanisms

To compensate for the variability observed between different flies, all S10b-follicles of a single fly (approximately 10–20 follicles) were divided into a control group and an experimental group. Inhibition and staining of living follicles, using either CFDA or DiBAC, were done simultaneously for 20 or 60 min, depending on the type of experiment, in R-14 medium containing the respective inhibitor and the fluorescent probe.

The following inhibitors of ion-transport mechanisms were used: Na^+^/H^+^-exchangers (NHE) and amiloride-sensitive Na^+^-channels were blocked with amiloride (Sigma-Aldrich, Germany; 10 μM; dissolved in DMSO), V-ATPases with bafilomycin A1 (Sigma-Aldrich; 160 nM; dissolved in DMSO), ATP-sensitive K^+^-channels with glibenclamide (Biomol, Germany; 100 μM; dissolved in DMSO), voltage-dependent L-type Ca^2+^-channels with verapamil-HCl (Sigma-Aldrich; 50 μM; dissolved in ethanol), Cl^−^-channels with 9-anthroic acid (Sigma-Aldrich; 100 μM; dissolved in ethanol) and Na^+^/K^+^/2Cl^−^-cotransporters with furosemide (Sigma-Aldrich; 1 mM; dissolved in DMSO). Control experiments were performed in R-14 medium containing 0.1–1% v/v ethanol or DMSO, respectively, without the inhibitor.

### Quantitative analysis of fluorescence intensities of CFDA and DiBAC

The original grey-scale images were used to measure the fluorescence intensities in the FCE with ImageJ (“mean grey value”).

#### WFM-experiment

WFM-images of each point of time were evaluated by measuring the columnar FCE of every single follicle. The mean grey values of the experimental group and the control group, respectively, were averaged (all follicles from the same fly). To compare the long-term effects of the inhibitors on either pH_i_ or V_mem_, the averaged values of each point of time were summed up and normalised. Three experiments for each inhibitor were included in this evaluation (*mean intensity ratio*).

#### SIM-experiment

SIM-images were used to measure individual regions of different types of FC: cFC, mbFC, pFC, dFC and vFC (see Fig. 1 a). All mean grey values of each FC type of the experimental group and the control group, respectively, were averaged for comparative analysis (all follicles from the same fly). For each inhibitor, at least five repetitions of the experiment were performed. The normalised values of the single experiments were merged into one evaluation (*relative intensity*).

Multiple t-tests with Holm-Sidak correction, Microsoft Excel and GraphPad Prism were used for statistical analysis. Graphpad Prism was also used for representation of the data.

## Additional files

Additional file 1: **Figure S1.** Development of pH_i_-gradients in the FCE during S8–12 (CFDA; SIM). Examples corresponding to Fig. 1b. **Figure S2.** Development of V_mem_-gradients in the FCE during S8–12 (DiBAC; SIM). Examples corresponding to Fig. 2b. **Figure S3.** Controls for pH_i_ (CFDA; WFM). Examples of S10b follicles incubated for 60 min in R-14 medium pH 5.5 (hydrochloric acid) and pH 8.0 (sodium hydroxide), respectively. (PPTX 9873 kb)

Additional file 2: **Table S1.** Development of pH_i_-gradients in the FCE during S8–12 (CFDA; SIM). Numerical values corresponding to Fig. 1 c. **Table S2.** Development of V_mem_-gradients in the FCE during S8–12 (DiBAC; SIM). Numerical values corresponding to Fig. 2 c. **Table S3.** Inhibitors of ion-transport mechanisms exert influence on the pH_i_ in the FCE during S10b (WFM-experiment; CFDA). Numerical values corresponding to Fig. 3a. **Table S4.** Inhibitors of ion-transport mechanisms exert influence on the V_mem_ in the FCE during S10b (WFM-experiment; DiBAC). Numerical values corresponding to Fig. 6a. **Table S5.** The a-p and d-v pH_i_-gradients in the FCE are affected by all inhibitors in S10b (SIM-experiment; CFDA). Numerical values corresponding to Figs. 4b and 5a. **Table S6.** The a-p V_mem_-gradient in the FCE is affected by most inhibitors in S10b. Some inhibitors exert influence on the d-v V_mem_-gradient (SIM-experiment; DiBAC). Numerical values corresponding to Figs. 7b and 8. (PPTX 78 kb)

## Data Availability

The datasets used during the current study are available from the corresponding author on reasonable request.

## **Abbrevations**

aFC: anterior follicle cells
a-p: antero-posterior
cFC: centripetal follicle cells
CFDA: 5-carboxyfluorescein diacetate, acetoxymethyl ester
DiBAC: bis-(1,3-dibutylbarbituric acid) trimethine oxonol
DIC: differential-interference contrast
DMSO: dimethyl sulfoxide
d-v: dorso-ventral
FC: follicle cells
FCE: follicle-cell epithelium
mbFC: mainbody follicle cells
NC: nurse cells
NHE: Na^+^/H^+^-exchangers
Oo: oocyte
pFC: posterior follicle cells
pH_i_: intracellular pH
S: stage
SIM: structured-illumination microscopy
V_mem_: membrane potential
WFM: wide-field microscopy
