# Supplementary material for: Electrochemical patterns during Drosophila oogenesis: ion-transport mechanisms generate stage-specific gradients of pH and membrane potential in the follicle-cell epithelium
Source: BMC Dev Biol. 2019 Jun 21;19:12. doi: 10.1186/s12861-019-0192-x (PMC6588877; doi:10.1186/s12861-019-0192-x)
Supplement: Supplementary file 1 — Figure S1. Development of pHi-gradients in the FCE during S8–12 (CFDA; SIM). Examples corresponding to Fig. 1b. Figure S2. Development of Vmem-gradients in the FCE during S8–12 (DiBAC; SIM). Examples corresponding to Fig. 2b. Figure S3. Controls for pHi (CFDA; WFM). Examples of S10b follicles incubated for 60 min in R-14 medium pH 5.5 (hydrochloric acid) and pH 8.0 (sodium hydroxide), respectively. (PPTX 9873 kb) [file 12861_2019_192_MOESM1_ESM.pptx]

## Slide 1
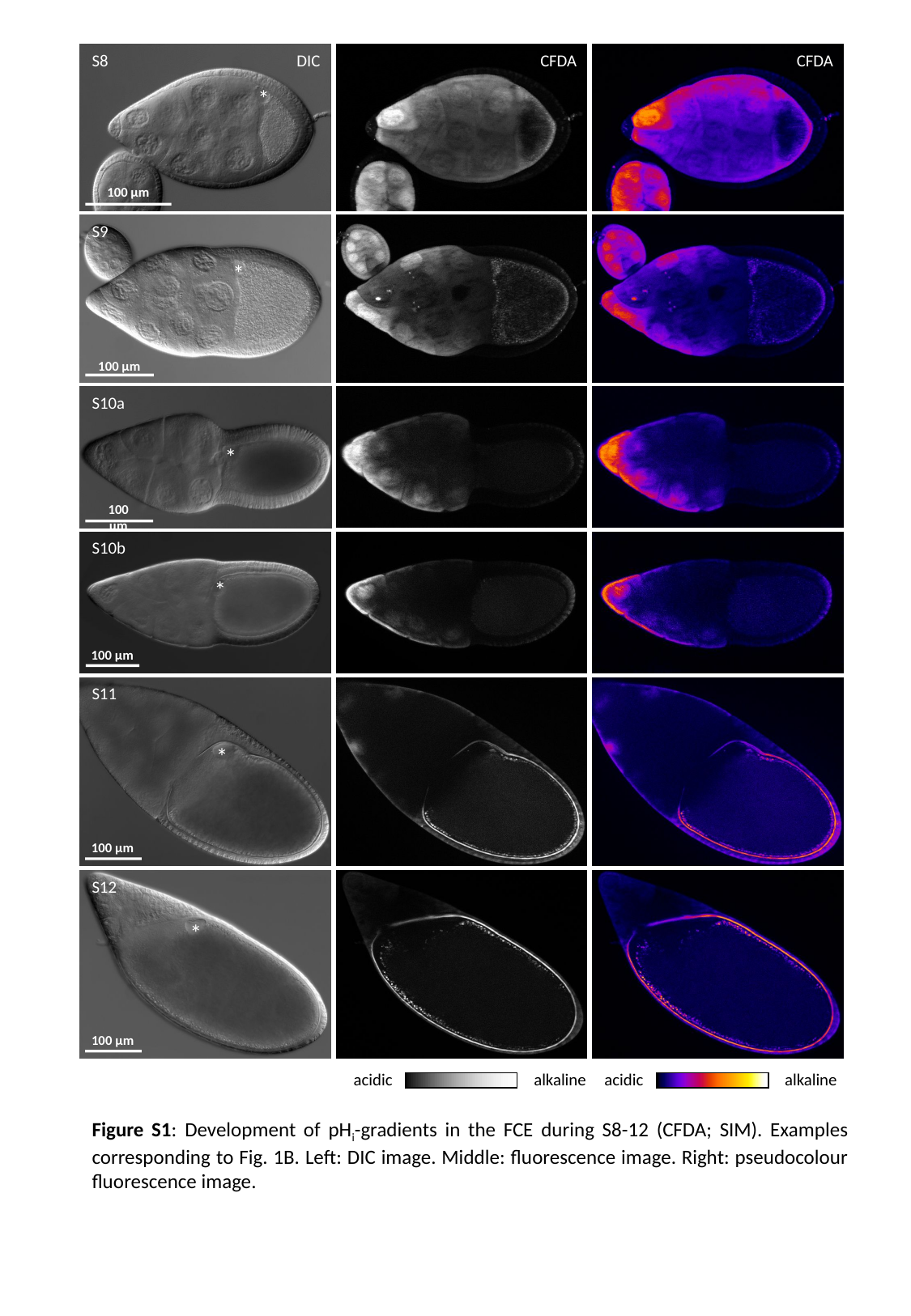

S8
DIC
CFDA
CFDA
*
100 µm
S9
*
100 µm
S10a
*
100 µm
DiBAC4(3)
(LUT)
S10b
*
100 µm
S11
*
100 µm
S12
*
100 µm
acidic
alkaline
acidic
alkaline
Figure S1: Development of pHi-gradients in the FCE during S8-12 (CFDA; SIM). Examples corresponding to Fig. 1B. Left: DIC image. Middle: fluorescence image. Right: pseudocolour fluorescence image.

## Slide 2
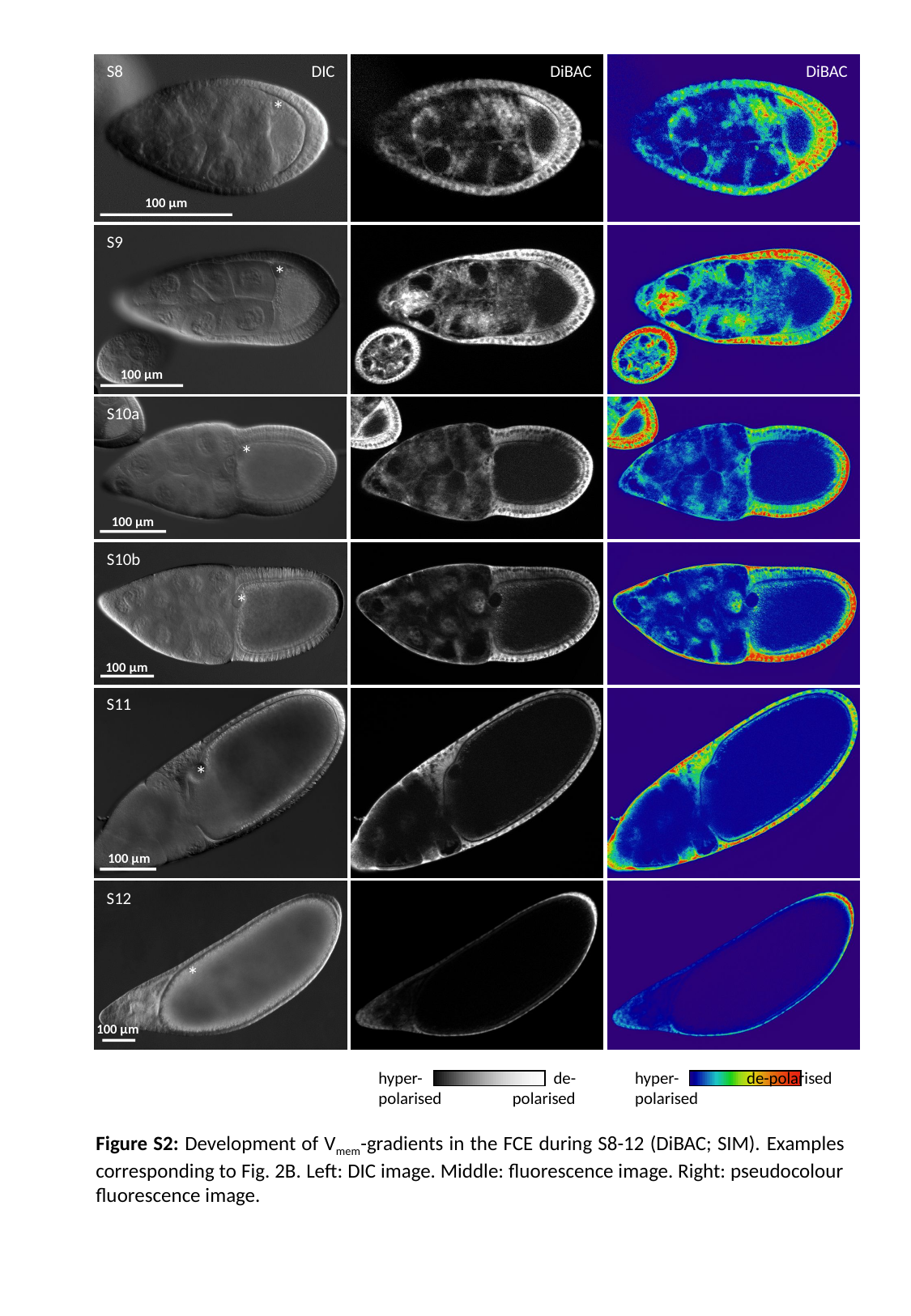

S8
DIC
DiBAC
DiBAC
*
100 µm
S9
*
100 µm
S10a
*
100 µm
DiBAC4(3)
(LUT)
S10b
*
100 µm
S11
*
100 µm
S12
*
100 µm
hyper-polarised
de-polarised
hyper-polarised
de-polarised
Figure S2: Development of Vmem-gradients in the FCE during S8-12 (DiBAC; SIM). Examples corresponding to Fig. 2B. Left: DIC image. Middle: fluorescence image. Right: pseudocolour fluorescence image.

## Slide 3
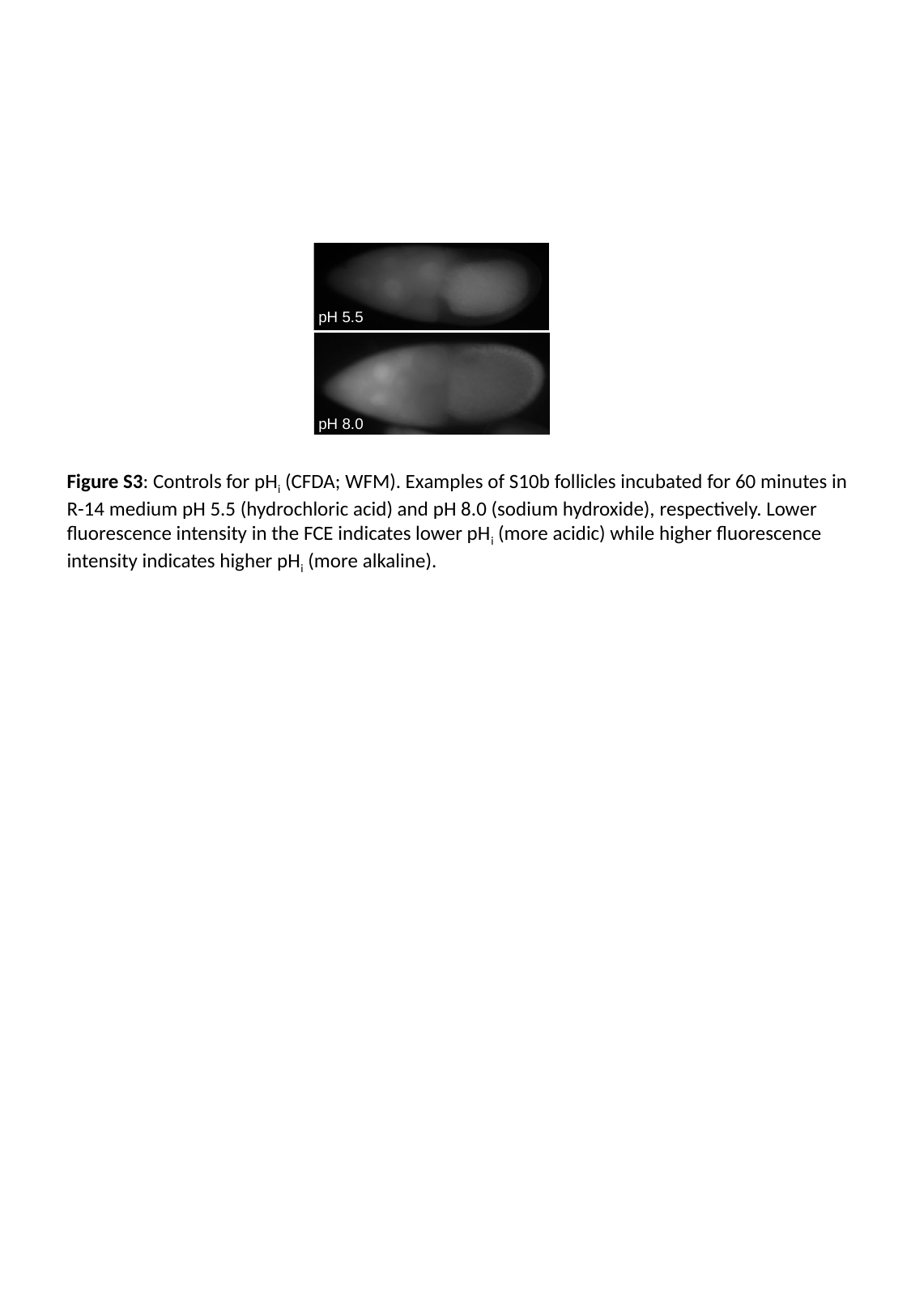

pH 5.5
pH 8.0
Figure S3: Controls for pHi (CFDA; WFM). Examples of S10b follicles incubated for 60 minutes in R-14 medium pH 5.5 (hydrochloric acid) and pH 8.0 (sodium hydroxide), respectively. Lower fluorescence intensity in the FCE indicates lower pHi (more acidic) while higher fluorescence intensity indicates higher pHi (more alkaline).
