# Supplementary material for: Electrochemical patterns during Drosophila oogenesis: ion-transport mechanisms generate stage-specific gradients of pH and membrane potential in the follicle-cell epithelium
Source: BMC Dev Biol. 2019 Jun 21;19:12. doi: 10.1186/s12861-019-0192-x (PMC6588877; doi:10.1186/s12861-019-0192-x)
Supplement: Supplementary file 2 — Table S1. Development of pHi-gradients in the FCE during S8–12 (CFDA; SIM). Numerical values corresponding to Fig. 1 c. Table S2. Development of Vmem-gradients in the FCE during S8–12 (DiBAC; SIM). Numerical values corresponding to Fig. 2 c. Table S3. Inhibitors of ion-transport mechanisms exert influence on the pHi in the FCE during S10b (WFM-experiment; CFDA). Numerical values corresponding to Fig. 3a. Table S4. Inhibitors of ion-transport mechanisms exert influence on the Vmem in the FCE during S10b (WFM-experiment; DiBAC). Numerical values corresponding to Fig. 6a. Table S5. The a-p and d-v pHi-gradients in the FCE are affected by all inhibitors in S10b (SIM-experiment; CFDA). Numerical values corresponding to Figs. 4b and 5a. Table S6. The a-p Vmem-gradient in the FCE is affected by most inhibitors in S10b. Some inhibitors exert influence on the d-v Vmem-gradient (SIM-experiment; DiBAC). Numerical values corresponding to Figs. 7b and 8. (PPTX 78 kb) [file 12861_2019_192_MOESM2_ESM.pptx]

## Slide 1
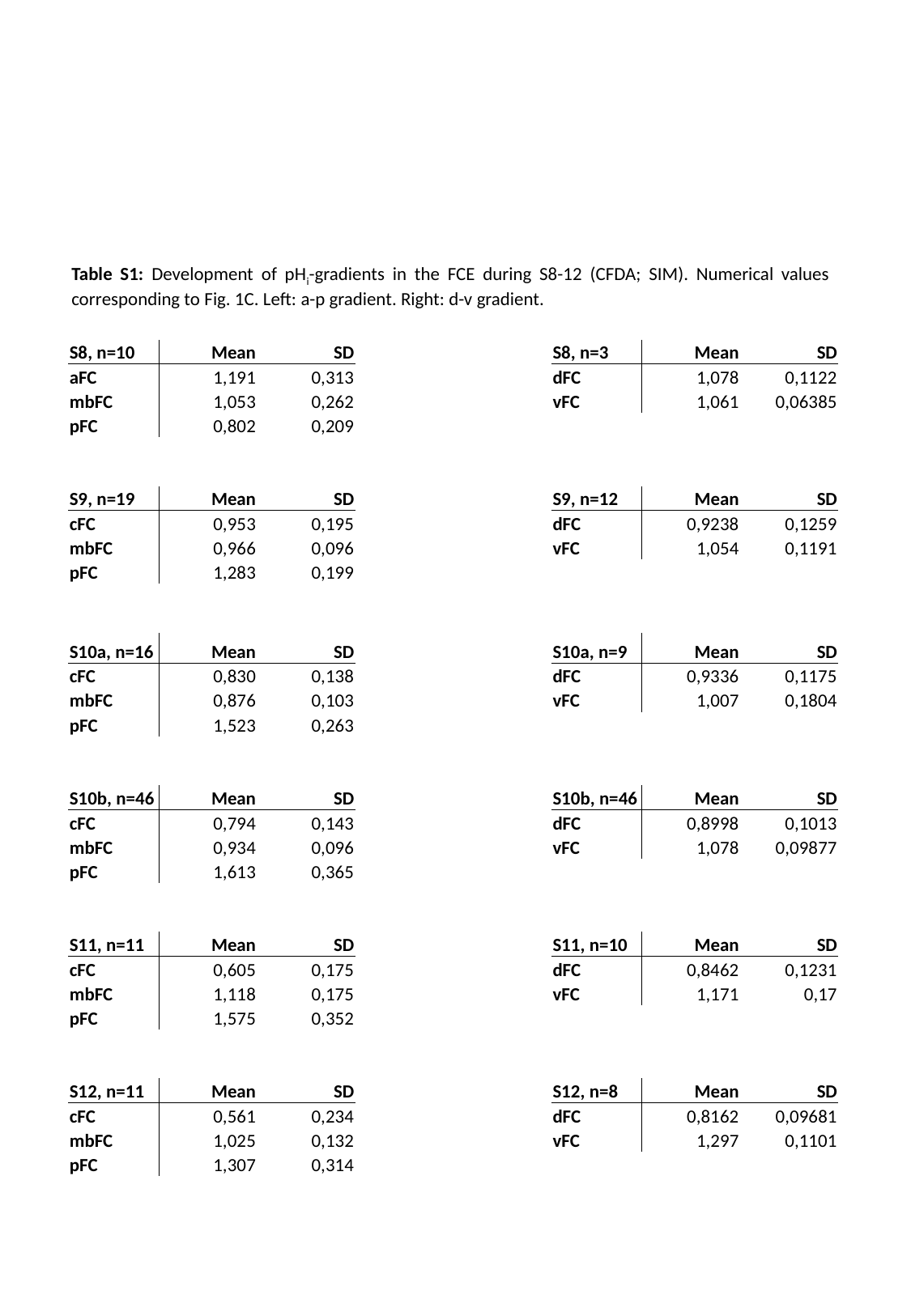

Table S1: Development of pHi-gradients in the FCE during S8-12 (CFDA; SIM). Numerical values corresponding to Fig. 1C. Left: a-p gradient. Right: d-v gradient.
| S8, n=10 | Mean | SD | | | S8, n=3 | Mean | SD |
| --- | --- | --- | --- | --- | --- | --- | --- |
| aFC | 1,191 | 0,313 | | | dFC | 1,078 | 0,1122 |
| mbFC | 1,053 | 0,262 | | | vFC | 1,061 | 0,06385 |
| pFC | 0,802 | 0,209 | | | | | |
| | | | | | | | |
| | | | | | | | |
| S9, n=19 | Mean | SD | | | S9, n=12 | Mean | SD |
| cFC | 0,953 | 0,195 | | | dFC | 0,9238 | 0,1259 |
| mbFC | 0,966 | 0,096 | | | vFC | 1,054 | 0,1191 |
| pFC | 1,283 | 0,199 | | | | | |
| | | | | | | | |
| | | | | | | | |
| S10a, n=16 | Mean | SD | | | S10a, n=9 | Mean | SD |
| cFC | 0,830 | 0,138 | | | dFC | 0,9336 | 0,1175 |
| mbFC | 0,876 | 0,103 | | | vFC | 1,007 | 0,1804 |
| pFC | 1,523 | 0,263 | | | | | |
| | | | | | | | |
| | | | | | | | |
| S10b, n=46 | Mean | SD | | | S10b, n=46 | Mean | SD |
| cFC | 0,794 | 0,143 | | | dFC | 0,8998 | 0,1013 |
| mbFC | 0,934 | 0,096 | | | vFC | 1,078 | 0,09877 |
| pFC | 1,613 | 0,365 | | | | | |
| | | | | | | | |
| | | | | | | | |
| S11, n=11 | Mean | SD | | | S11, n=10 | Mean | SD |
| cFC | 0,605 | 0,175 | | | dFC | 0,8462 | 0,1231 |
| mbFC | 1,118 | 0,175 | | | vFC | 1,171 | 0,17 |
| pFC | 1,575 | 0,352 | | | | | |
| | | | | | | | |
| | | | | | | | |
| S12, n=11 | Mean | SD | | | S12, n=8 | Mean | SD |
| cFC | 0,561 | 0,234 | | | dFC | 0,8162 | 0,09681 |
| mbFC | 1,025 | 0,132 | | | vFC | 1,297 | 0,1101 |
| pFC | 1,307 | 0,314 | | | | | |

## Slide 2
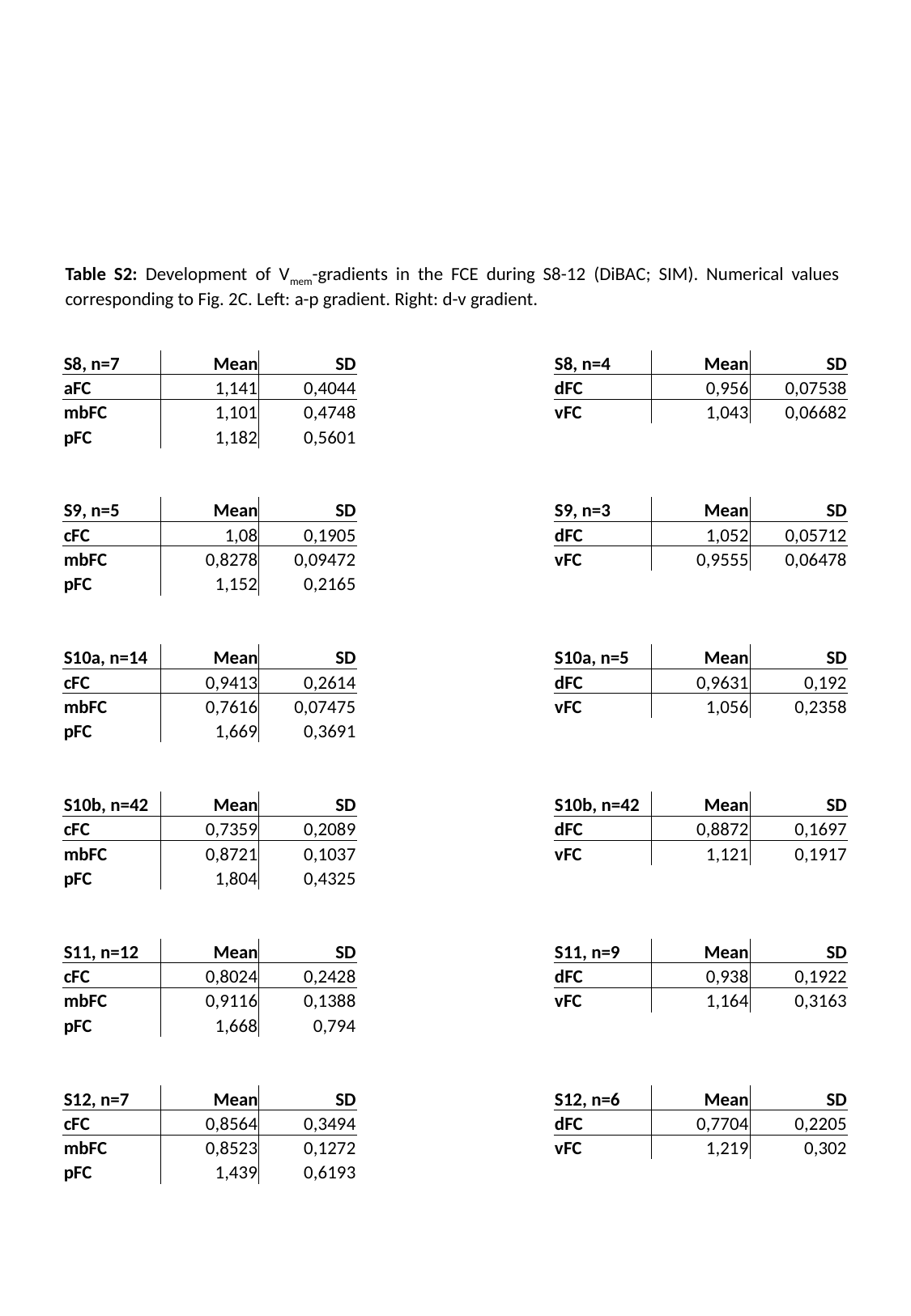

Table S2: Development of Vmem-gradients in the FCE during S8-12 (DiBAC; SIM). Numerical values corresponding to Fig. 2C. Left: a-p gradient. Right: d-v gradient.
| S8, n=7 | Mean | SD | | | S8, n=4 | Mean | SD |
| --- | --- | --- | --- | --- | --- | --- | --- |
| aFC | 1,141 | 0,4044 | | | dFC | 0,956 | 0,07538 |
| mbFC | 1,101 | 0,4748 | | | vFC | 1,043 | 0,06682 |
| pFC | 1,182 | 0,5601 | | | | | |
| | | | | | | | |
| | | | | | | | |
| S9, n=5 | Mean | SD | | | S9, n=3 | Mean | SD |
| cFC | 1,08 | 0,1905 | | | dFC | 1,052 | 0,05712 |
| mbFC | 0,8278 | 0,09472 | | | vFC | 0,9555 | 0,06478 |
| pFC | 1,152 | 0,2165 | | | | | |
| | | | | | | | |
| | | | | | | | |
| S10a, n=14 | Mean | SD | | | S10a, n=5 | Mean | SD |
| cFC | 0,9413 | 0,2614 | | | dFC | 0,9631 | 0,192 |
| mbFC | 0,7616 | 0,07475 | | | vFC | 1,056 | 0,2358 |
| pFC | 1,669 | 0,3691 | | | | | |
| | | | | | | | |
| | | | | | | | |
| S10b, n=42 | Mean | SD | | | S10b, n=42 | Mean | SD |
| cFC | 0,7359 | 0,2089 | | | dFC | 0,8872 | 0,1697 |
| mbFC | 0,8721 | 0,1037 | | | vFC | 1,121 | 0,1917 |
| pFC | 1,804 | 0,4325 | | | | | |
| | | | | | | | |
| | | | | | | | |
| S11, n=12 | Mean | SD | | | S11, n=9 | Mean | SD |
| cFC | 0,8024 | 0,2428 | | | dFC | 0,938 | 0,1922 |
| mbFC | 0,9116 | 0,1388 | | | vFC | 1,164 | 0,3163 |
| pFC | 1,668 | 0,794 | | | | | |
| | | | | | | | |
| | | | | | | | |
| S12, n=7 | Mean | SD | | | S12, n=6 | Mean | SD |
| cFC | 0,8564 | 0,3494 | | | dFC | 0,7704 | 0,2205 |
| mbFC | 0,8523 | 0,1272 | | | vFC | 1,219 | 0,302 |
| pFC | 1,439 | 0,6193 | | | | | |

## Slide 3
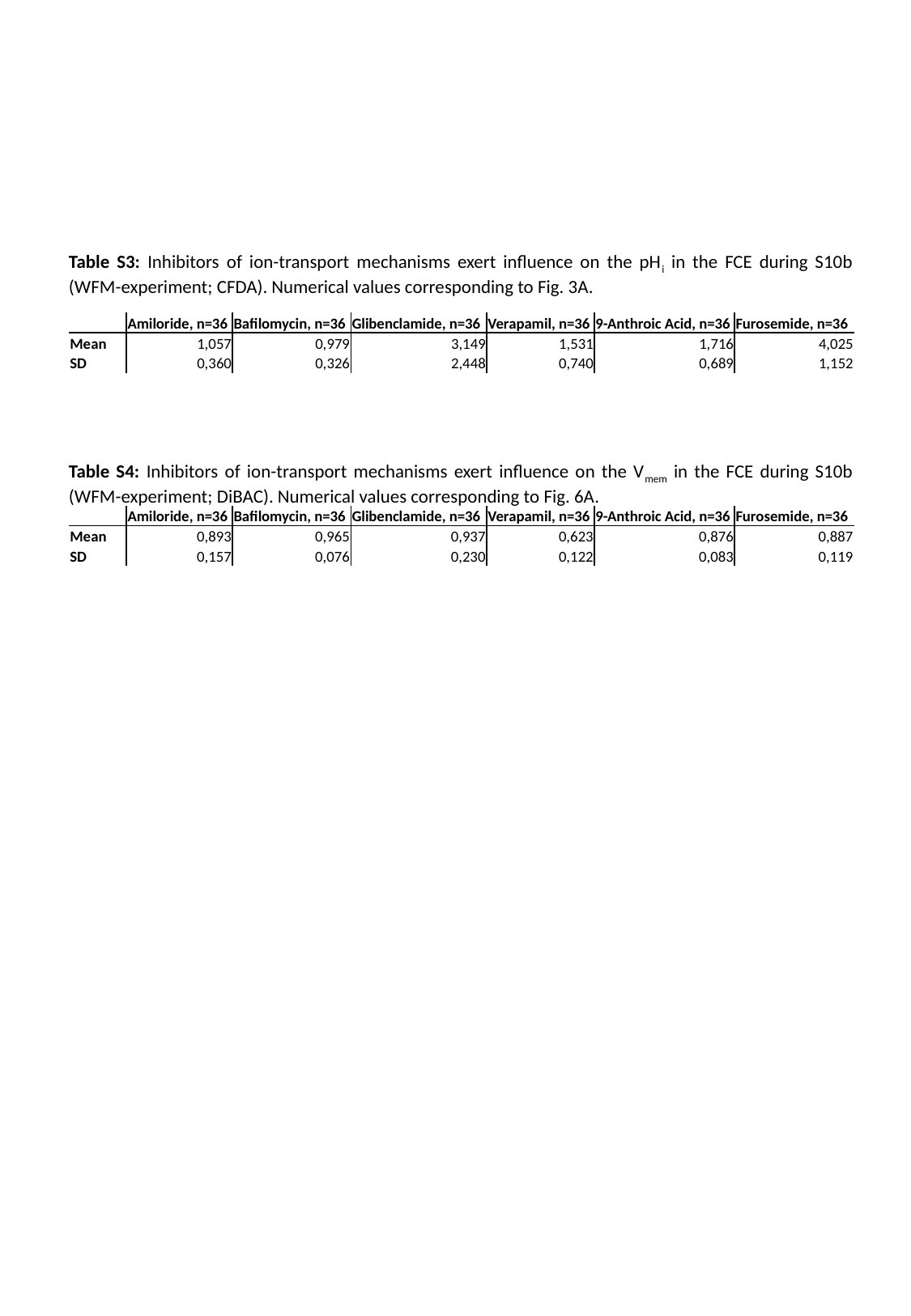

Table S3: Inhibitors of ion-transport mechanisms exert influence on the pHi in the FCE during S10b (WFM-experiment; CFDA). Numerical values corresponding to Fig. 3A.
| | Amiloride, n=36 | Bafilomycin, n=36 | Glibenclamide, n=36 | Verapamil, n=36 | 9-Anthroic Acid, n=36 | Furosemide, n=36 |
| --- | --- | --- | --- | --- | --- | --- |
| Mean | 1,057 | 0,979 | 3,149 | 1,531 | 1,716 | 4,025 |
| SD | 0,360 | 0,326 | 2,448 | 0,740 | 0,689 | 1,152 |
| | | | | | | |
| | | | | | | |
| | | | | | | |
| | Amiloride, n=36 | Bafilomycin, n=36 | Glibenclamide, n=36 | Verapamil, n=36 | 9-Anthroic Acid, n=36 | Furosemide, n=36 |
| Mean | 0,893 | 0,965 | 0,937 | 0,623 | 0,876 | 0,887 |
| SD | 0,157 | 0,076 | 0,230 | 0,122 | 0,083 | 0,119 |
Table S4: Inhibitors of ion-transport mechanisms exert influence on the Vmem in the FCE during S10b (WFM-experiment; DiBAC). Numerical values corresponding to Fig. 6A.

## Slide 4
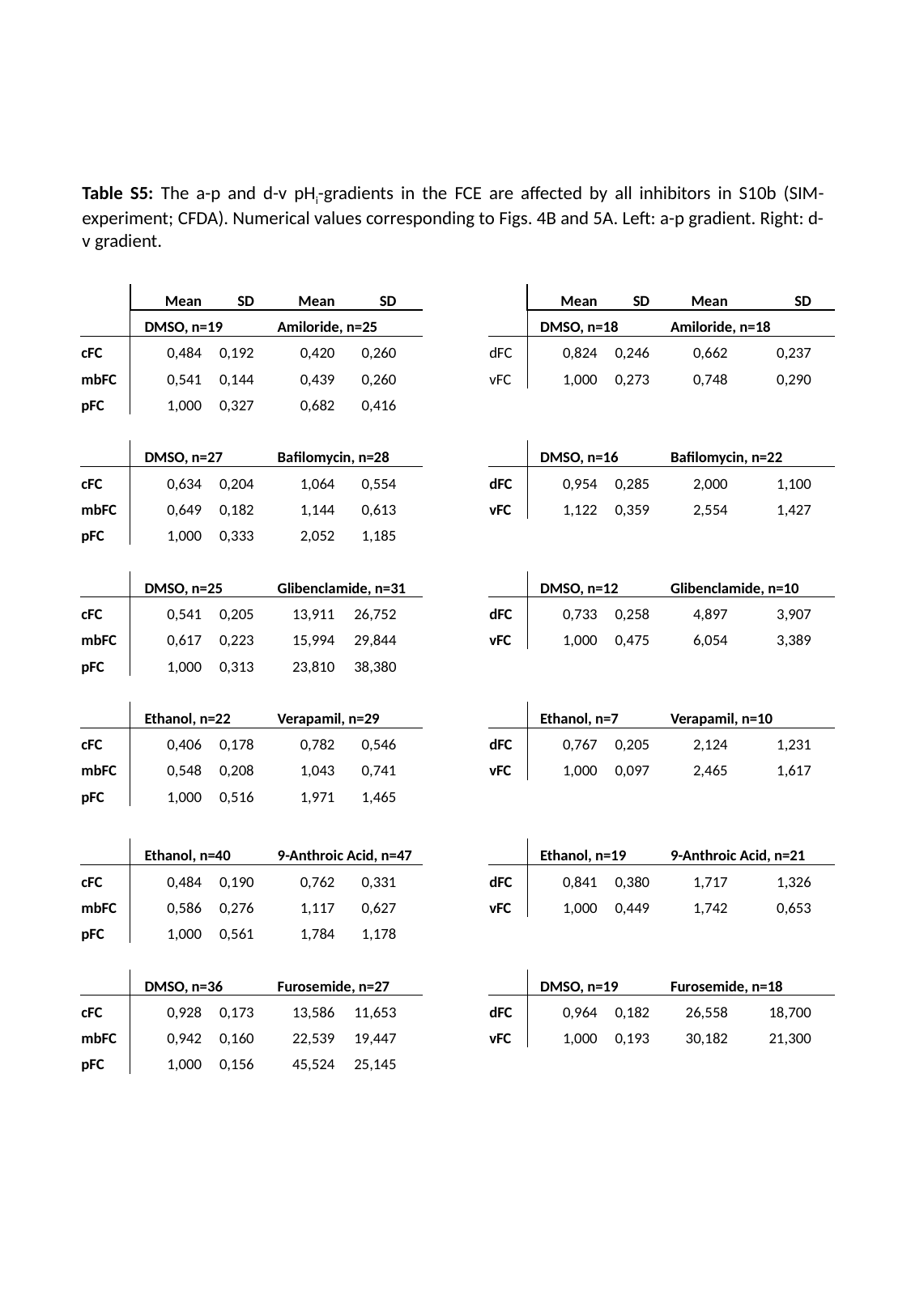

Table S5: The a-p and d-v pHi-gradients in the FCE are affected by all inhibitors in S10b (SIM-experiment; CFDA). Numerical values corresponding to Figs. 4B and 5A. Left: a-p gradient. Right: d-v gradient.
| | | Mean | SD | | Mean | SD | | | | | Mean | SD | | Mean | SD | | |
| --- | --- | --- | --- | --- | --- | --- | --- | --- | --- | --- | --- | --- | --- | --- | --- | --- | --- |
| | | DMSO, n=19 | | | Amiloride, n=25 | | | | | | DMSO, n=18 | | | Amiloride, n=18 | | | |
| cFC | | 0,484 | 0,192 | | 0,420 | 0,260 | | | dFC | | 0,824 | 0,246 | | 0,662 | 0,237 | | |
| mbFC | | 0,541 | 0,144 | | 0,439 | 0,260 | | | vFC | | 1,000 | 0,273 | | 0,748 | 0,290 | | |
| pFC | | 1,000 | 0,327 | | 0,682 | 0,416 | | | | | | | | | | | |
| | | | | | | | | | | | | | | | | | |
| | | DMSO, n=27 | | | Bafilomycin, n=28 | | | | | | DMSO, n=16 | | | Bafilomycin, n=22 | | | |
| cFC | | 0,634 | 0,204 | | 1,064 | 0,554 | | | dFC | | 0,954 | 0,285 | | 2,000 | 1,100 | | |
| mbFC | | 0,649 | 0,182 | | 1,144 | 0,613 | | | vFC | | 1,122 | 0,359 | | 2,554 | 1,427 | | |
| pFC | | 1,000 | 0,333 | | 2,052 | 1,185 | | | | | | | | | | | |
| | | | | | | | | | | | | | | | | | |
| | | DMSO, n=25 | | | Glibenclamide, n=31 | | | | | | DMSO, n=12 | | | Glibenclamide, n=10 | | | |
| cFC | | 0,541 | 0,205 | | 13,911 | 26,752 | | | dFC | | 0,733 | 0,258 | | 4,897 | 3,907 | | |
| mbFC | | 0,617 | 0,223 | | 15,994 | 29,844 | | | vFC | | 1,000 | 0,475 | | 6,054 | 3,389 | | |
| pFC | | 1,000 | 0,313 | | 23,810 | 38,380 | | | | | | | | | | | |
| | | | | | | | | | | | | | | | | | |
| | | Ethanol, n=22 | | | Verapamil, n=29 | | | | | | Ethanol, n=7 | | | Verapamil, n=10 | | | |
| cFC | | 0,406 | 0,178 | | 0,782 | 0,546 | | | dFC | | 0,767 | 0,205 | | 2,124 | 1,231 | | |
| mbFC | | 0,548 | 0,208 | | 1,043 | 0,741 | | | vFC | | 1,000 | 0,097 | | 2,465 | 1,617 | | |
| pFC | | 1,000 | 0,516 | | 1,971 | 1,465 | | | | | | | | | | | |
| | | | | | | | | | | | | | | | | | |
| | | Ethanol, n=40 | | | 9-Anthroic Acid, n=47 | | | | | | Ethanol, n=19 | | | 9-Anthroic Acid, n=21 | | | |
| cFC | | 0,484 | 0,190 | | 0,762 | 0,331 | | | dFC | | 0,841 | 0,380 | | 1,717 | 1,326 | | |
| mbFC | | 0,586 | 0,276 | | 1,117 | 0,627 | | | vFC | | 1,000 | 0,449 | | 1,742 | 0,653 | | |
| pFC | | 1,000 | 0,561 | | 1,784 | 1,178 | | | | | | | | | | | |
| | | | | | | | | | | | | | | | | | |
| | | DMSO, n=36 | | | Furosemide, n=27 | | | | | | DMSO, n=19 | | | Furosemide, n=18 | | | |
| cFC | | 0,928 | 0,173 | | 13,586 | 11,653 | | | dFC | | 0,964 | 0,182 | | 26,558 | 18,700 | | |
| mbFC | | 0,942 | 0,160 | | 22,539 | 19,447 | | | vFC | | 1,000 | 0,193 | | 30,182 | 21,300 | | |
| pFC | | 1,000 | 0,156 | | 45,524 | 25,145 | | | | | | | | | | | |

## Slide 5
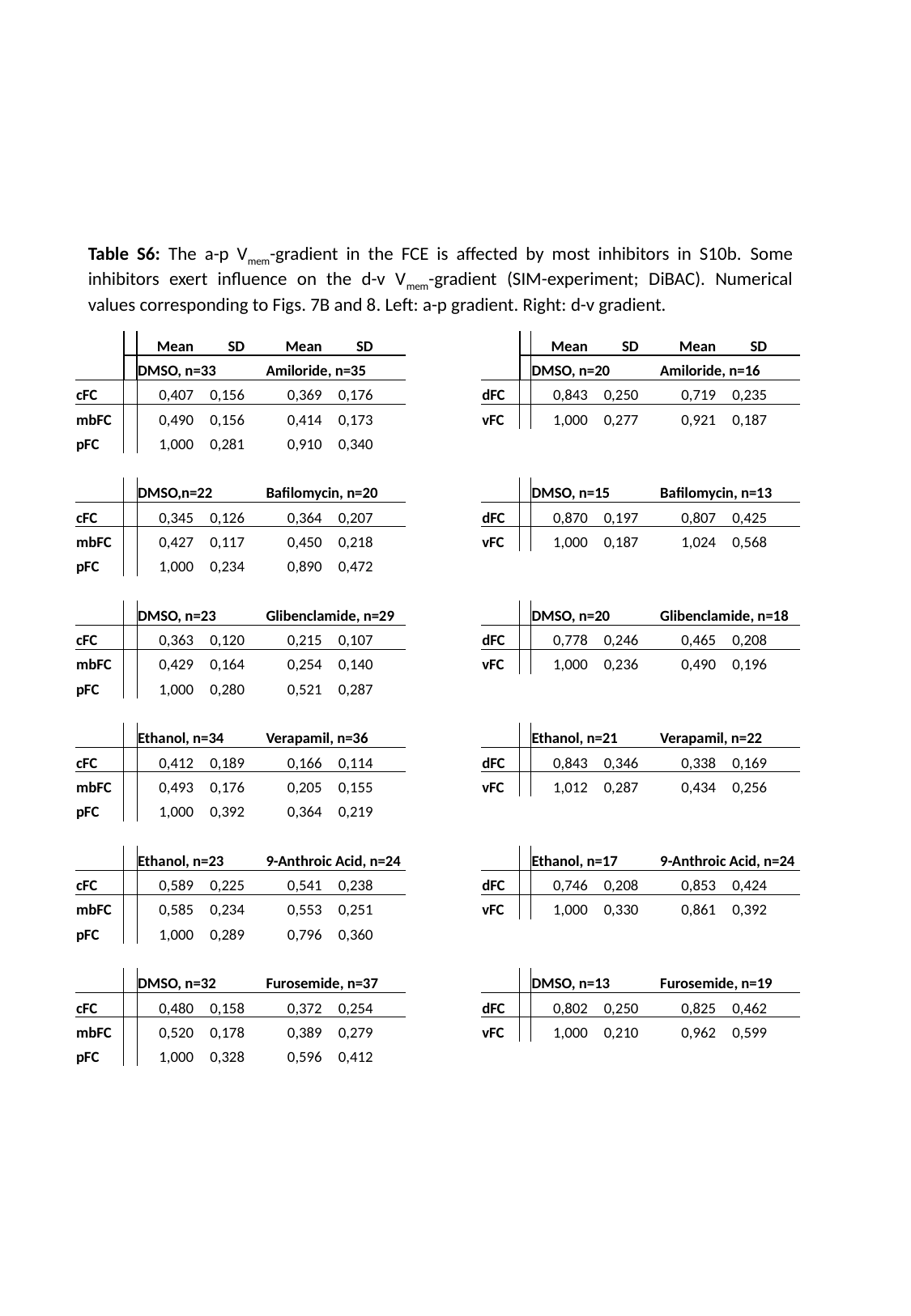

Table S6: The a-p Vmem-gradient in the FCE is affected by most inhibitors in S10b. Some inhibitors exert influence on the d-v Vmem-gradient (SIM-experiment; DiBAC). Numerical values corresponding to Figs. 7B and 8. Left: a-p gradient. Right: d-v gradient.
| | | Mean | SD | | Mean | SD | | | | | | Mean | SD | | Mean | SD | | |
| --- | --- | --- | --- | --- | --- | --- | --- | --- | --- | --- | --- | --- | --- | --- | --- | --- | --- | --- |
| | | DMSO, n=33 | | | Amiloride, n=35 | | | | | | | DMSO, n=20 | | | Amiloride, n=16 | | | |
| cFC | | 0,407 | 0,156 | | 0,369 | 0,176 | | | | dFC | | 0,843 | 0,250 | | 0,719 | 0,235 | | |
| mbFC | | 0,490 | 0,156 | | 0,414 | 0,173 | | | | vFC | | 1,000 | 0,277 | | 0,921 | 0,187 | | |
| pFC | | 1,000 | 0,281 | | 0,910 | 0,340 | | | | | | | | | | | | |
| | | | | | | | | | | | | | | | | | | |
| | | DMSO,n=22 | | | Bafilomycin, n=20 | | | | | | | DMSO, n=15 | | | Bafilomycin, n=13 | | | |
| cFC | | 0,345 | 0,126 | | 0,364 | 0,207 | | | | dFC | | 0,870 | 0,197 | | 0,807 | 0,425 | | |
| mbFC | | 0,427 | 0,117 | | 0,450 | 0,218 | | | | vFC | | 1,000 | 0,187 | | 1,024 | 0,568 | | |
| pFC | | 1,000 | 0,234 | | 0,890 | 0,472 | | | | | | | | | | | | |
| | | | | | | | | | | | | | | | | | | |
| | | DMSO, n=23 | | | Glibenclamide, n=29 | | | | | | | DMSO, n=20 | | | Glibenclamide, n=18 | | | |
| cFC | | 0,363 | 0,120 | | 0,215 | 0,107 | | | | dFC | | 0,778 | 0,246 | | 0,465 | 0,208 | | |
| mbFC | | 0,429 | 0,164 | | 0,254 | 0,140 | | | | vFC | | 1,000 | 0,236 | | 0,490 | 0,196 | | |
| pFC | | 1,000 | 0,280 | | 0,521 | 0,287 | | | | | | | | | | | | |
| | | | | | | | | | | | | | | | | | | |
| | | Ethanol, n=34 | | | Verapamil, n=36 | | | | | | | Ethanol, n=21 | | | Verapamil, n=22 | | | |
| cFC | | 0,412 | 0,189 | | 0,166 | 0,114 | | | | dFC | | 0,843 | 0,346 | | 0,338 | 0,169 | | |
| mbFC | | 0,493 | 0,176 | | 0,205 | 0,155 | | | | vFC | | 1,012 | 0,287 | | 0,434 | 0,256 | | |
| pFC | | 1,000 | 0,392 | | 0,364 | 0,219 | | | | | | | | | | | | |
| | | | | | | | | | | | | | | | | | | |
| | | Ethanol, n=23 | | | 9-Anthroic Acid, n=24 | | | | | | | Ethanol, n=17 | | | 9-Anthroic Acid, n=24 | | | |
| cFC | | 0,589 | 0,225 | | 0,541 | 0,238 | | | | dFC | | 0,746 | 0,208 | | 0,853 | 0,424 | | |
| mbFC | | 0,585 | 0,234 | | 0,553 | 0,251 | | | | vFC | | 1,000 | 0,330 | | 0,861 | 0,392 | | |
| pFC | | 1,000 | 0,289 | | 0,796 | 0,360 | | | | | | | | | | | | |
| | | | | | | | | | | | | | | | | | | |
| | | DMSO, n=32 | | | Furosemide, n=37 | | | | | | | DMSO, n=13 | | | Furosemide, n=19 | | | |
| cFC | | 0,480 | 0,158 | | 0,372 | 0,254 | | | | dFC | | 0,802 | 0,250 | | 0,825 | 0,462 | | |
| mbFC | | 0,520 | 0,178 | | 0,389 | 0,279 | | | | vFC | | 1,000 | 0,210 | | 0,962 | 0,599 | | |
| pFC | | 1,000 | 0,328 | | 0,596 | 0,412 | | | | | | | | | | | | |
